# Chromosome-scale *Echinococcus granulosus* (genotype G1) genome reveals the *Eg*95 gene family and conservation of the EG95-vaccine molecule

**DOI:** 10.1038/s42003-022-03125-1

**Published:** 2022-03-03

**Authors:** Pasi K. Korhonen, Liina Kinkar, Neil D. Young, Huimin Cai, Marshall W. Lightowlers, Charles Gauci, Abdul Jabbar, Bill C. H. Chang, Tao Wang, Andreas Hofmann, Anson V. Koehler, Junhua Li, Jiandong Li, Daxi Wang, Jiefang Yin, Huanming Yang, David J. Jenkins, Urmas Saarma, Teivi Laurimäe, Mohammad Rostami-Nejad, Malik Irshadullah, Hossein Mirhendi, Mitra Sharbatkhori, Francisco Ponce-Gordo, Sami Simsek, Adriano Casulli, Houria Zait, Hripsime Atoyan, Mario Luiz de la Rue, Thomas Romig, Marion Wassermann, Sargis A. Aghayan, Hasmik Gevorgyan, Bicheng Yang, Robin B. Gasser

**Affiliations:** 1 Department of Veterinary Biosciences, Melbourne Veterinary School, Faculty of Veterinary and Agricultural Sciences, The University of Melbourne, Parkville, VIC 3010 Australia; 2 BGI-Shenzhen, Shenzhen, 518083 China; 3 Shenzhen Key Laboratory of Unknown Pathogen Identification, BGI-Shenzhen, Shenzhen, 518083 China; 4 School of Animal and Veterinary Sciences, Charles Sturt University, Locked Bag 588, Wagga Wagga, NSW 2678 Australia; 5 Department of Zoology, Institute of Ecology and Earth Sciences, University of Tartu, J. Liivi 2, Tartu, 50409 Estonia; 6 Gastroenterology and Liver Diseases Research Center, Research Institute for Gastroenterology and Liver Diseases, Shahid Beheshti University of Medical Sciences, Tehran, Iran; 7 Section of Parasitology, Department of Zoology, Aligarh Muslim University, Aligarh, 202002 India; 8 Department of Medical Parasitology and Mycology, School of Medicine, Isfahan University of Medical Sciences, Isfahan, Iran; 9 Laboratory Sciences Research Center, Golestan University of Medical Sciences, Gorgan, Iran; 10 Department of Parasitology, Faculty of Pharmacy, Complutense University, Plaza Ramón y Cajal s/n, 28040 Madrid, Spain; 11 Department of Parasitology, Faculty of Veterinary Medicine, University of Firat, 23119 Elazig, Turkey; 12 World Health Organization Collaborating Centre for the Epidemiology, Detection and Control of Cystic and Alveolar Echinococcosis, European Union Reference Laboratory for Parasites (EURLP), Istituto Superiore di Sanità, Viale Regina Elena 299, 00161 Rome, Italy; 13 Parasitology and Mycology Department, Mustapha University Hospital, 16000 Algiers, Algeria; 14 Yerevan State University, Department of Zoology, Alex Manoogian, Yerevan, 0025 Armenia; 15 Universidade Federal de Santa Maria, Departamento de Microbiologia e Parasitologia, Santa Maria, RS Brazil; 16 Institute of Zoology, Parasitology Unit, University of Hohenheim, 70599 Stuttgart, Germany; 17 Chair of Zoology, Yerevan State University, 1 Alex Manoogian, Yerevan, 0025 Armenia; 18 Molecular Parasitology Research Group, Scientific Center – Zoology and Hydroecology, 7P. Sevak str, Yerevan, 0014 Armenia; 19 BGI Australia, Oceania, BGI Group, CBCRB Building, Herston Road, Herston, QLD 4006 Australia

**Keywords:** Parasitology, Microbiology

## Abstract

Cystic echinococcosis is a socioeconomically important parasitic disease caused by the larval stage of the canid tapeworm *Echinococcus granulosus*, afflicting millions of humans and animals worldwide. The development of a vaccine (called EG95) has been the most notable translational advance in the fight against this disease in animals. However, almost nothing is known about the genomic organisation/location of the family of genes encoding EG95 and related molecules, the extent of their conservation or their functions. The lack of a complete reference genome for *E. granulosus* genotype G1 has been a major obstacle to addressing these areas. Here, we assembled a chromosomal-scale genome for this genotype by scaffolding to a high quality genome for the congener *E. multilocularis*, localised *Eg*95 gene family members in this genome, and evaluated the conservation of the EG95 vaccine molecule. These results have marked implications for future explorations of aspects such as developmentally-regulated gene transcription/expression (using replicate samples) for all *E. granulosus* stages; structural and functional roles of non-coding genome regions; molecular ‘cross-talk’ between oncosphere and the immune system; and defining the precise function(s) of EG95. Applied aspects should include developing improved tools for the diagnosis and chemotherapy of cystic echinococcosis of humans.

## Abstract (web summary)

A high-quality genome for the parasitic tapeworm, *Echinococcus granulosus*, provides further insight into the EG95 vaccine target for cystic echinococcosis.

## Introduction

Cystic echinococcosis (hydatidosis) of humans is a neglected tropical disease (NTD) caused by the larval (metacestode) stage of the tapeworm (cestode) *Echinococcus granulosus* (family Taeniidae). This parasite has a complex life cycle, involving definitive hosts (canids) and intermediate hosts (ungulates—such as sheep, goats, cattle, camels—and macropods). Humans become infected when they accidentally ingest eggs released from canids infected with adult tapeworms; motile larvae (oncospheres) hatch from these eggs, penetrate the intestinal wall, enter blood vessels and are then passively transported to key predilection sites, mostly liver and lung. There, oncospheres grow and develop to cysts (over months and years), which internally produce larval stages (protoscoleces) asexually. The growth and propagation of these cysts cause severe disease^1^.

The prevention and control of echinococcosis rely on breaking transmission from host to host. Although canid definitive hosts can be treated at regular intervals (3–4 weeks) to eliminate adult worms from their small intestines, chemotherapy of people affected by echinococcosis with drugs, such as mebendazole or albendazole, is often ineffective^2,3^. An effective means of control is to vaccinate intermediate hosts (e.g., sheep) against *E. granulosus* to prevent them from becoming infected, thus disrupting transmission to definitive hosts^4–6^. Indeed, the development of a recombinant vaccine (called EG95) that protects sheep (with an efficacy of 95–99%) against echinococcosis^6,7^ has been one of the biggest milestones in the fight against neglected tropical diseases (cestodiases) caused by taeniid cestodes^4,8,9^.

Despite this breakthrough and the major relevance of this vaccine molecule, there is limited information regarding the genome organisation and fundamental biological role(s) of genes encoding EG95 as well as their conservation/variability and immunobiology. Using classical molecular approaches, key studies revealed that EG95 (encoded by a gene originally called *eg*95-1) was represented by a family of genes and a pseudogene^10,11^, and gene products have been localised specifically to the penetration glands (type-1) of the infective larval (oncosphere) stage^12^. Recent work^13^ attempted to localise the *Eg*95 genes in the genome, but did not achieve an outcome because of the fragmentation of draft genomes for *E. granulosus* (genotype G1) available at the time of investigation, leading to an inability to reliably map the coding genes to these genomes^13^. Currently available draft genomes for *E. granulosus*^14,16^ were assembled from short-read data sets, produced using a ‘second generation’, high throughput sequencing platform (Illumina technology), and one assembly was guided by scaffolding to a well-assembled genome for the congener *E. multilocularis*^14^. However, the use of short-read data sets does not allow the accurate assembly of long repeat regions within cestode genomes^15^, preventing the characterisation of non-coding RNA genes and some gene families within such regions, such as that encoding EG95. This challenge can be overcome by using third-generation, long-read sequencing technologies^15^. Thus, there has been a major need for a genome of near-chromosomal contiguity to enable (i) the accurate mapping of genes to the genome; (ii) the exploration of the organisation of the *Eg*95 and other gene families; (iii) fundamental investigations of the molecular biology, biochemistry and genetics of *E. granulosus*; and (iv) the development of improved diagnostic tools and new chemotherapies against cystic echinococcosis in humans. Here, we employed a combination of sequencing methods to generate complementary data sets to achieve a high-quality (chromosome-scale) genome assembly for genotype G1 of *E. granulosus*, localised members of the *Eg*95 gene family in this genome and assessed sequence conservation of the EG95 vaccine molecule. We discuss the implications of this study for future research on *Echinococcus*/echinococcosis.

## Results and discussion

### Assembly of the *Eg*-G1s reference genome

**Fig. 1:**
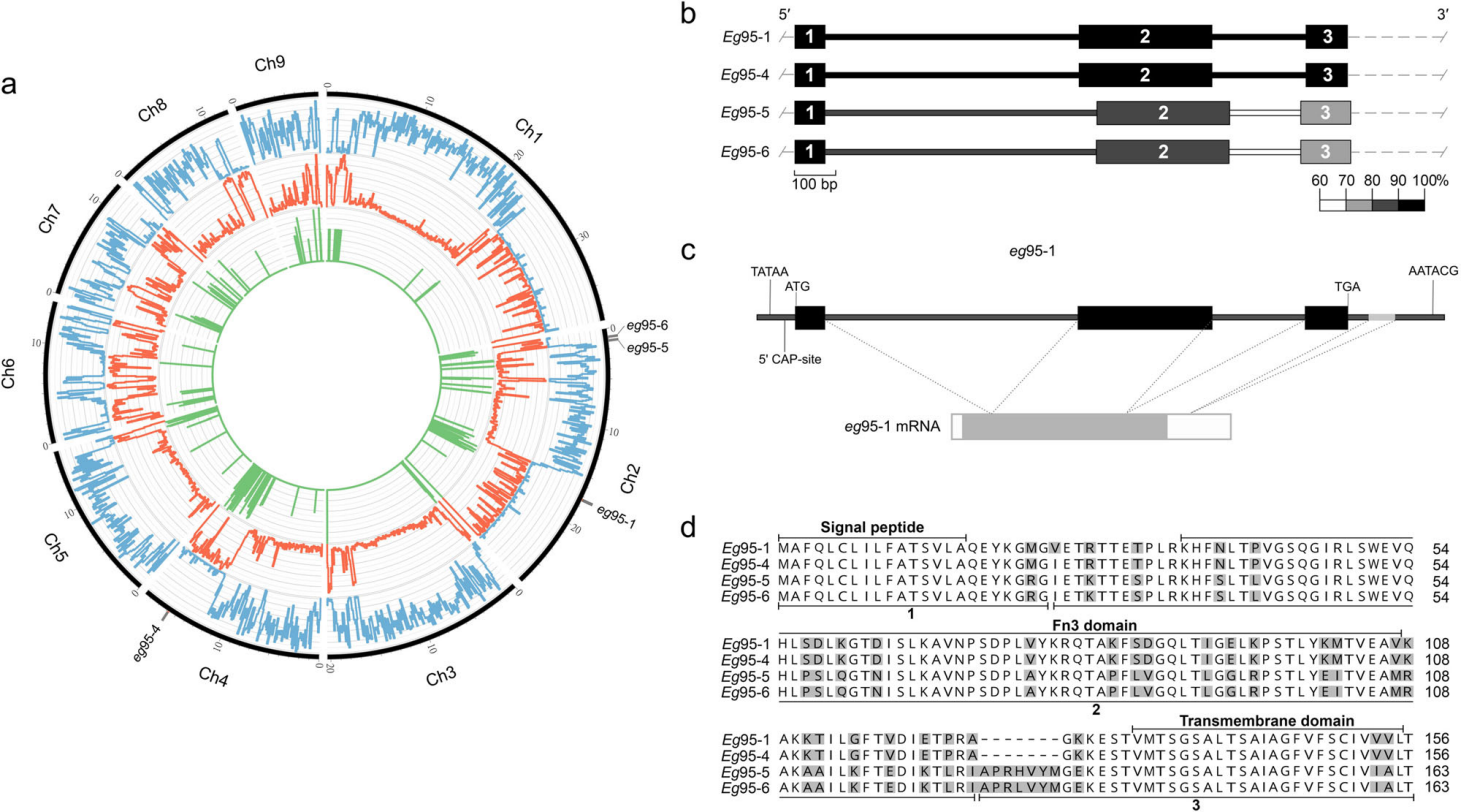
The genome *Eg*-G1s of *Echinococcus granulosus* (genotype G1) and the *Eg*95 gene family. **a** Circular representation of the *Echinococcus granulosus* genome (genotype G1; designated *Eg-*G1s) with nine chromosomes (Ch1 to Ch9); indicated are gene (blue), repeat (orange) and encoded RNA (green; log_2_) densities ranging from 0 to 100% (bin-size of 100 kb) across the genome and the locations of the four *Eg*95 genes (*Eg*95-1, -4, -5 and -6). **b** Structure of the four *Eg*95 gene family members—thick and thin bars denote 3 exons and 2 introns, respectively. Black bars indicate 100% identity to *Eg*95-1; shades of grey to white correspond to sequence identity (%) to *Eg*95-1 (scale, below). **c** Structure of the *Eg*95-1 gene and mRNA. Predicted Goldberg-Hogness box (TATAA), start site (ATG), termination codon (TGA) and polyadenylation signal (AATACG) are indicated; the first and last exons are flanked by non-coding regions; mRNA includes 5ʹ- and 3ʹ-UTRs (white) and coding regions (grey). **d** Complete amino acid sequence of EG95-1 compared with those predicted for EG95-4, EG95-5 and EG95-6. Dashes indicate gaps inserted for the purpose of the alignment. Pairwise sequence comparisons among these four sequences range from ~77% to 99% identity.

A high-quality reference genome for *E. granulosus* (genotype G1) was essential to undertake structural and comparative genomic analyses. The long-read data obtained (31.7 Gb, 212-fold coverage; Supplementary Table 1) were combined with available paired-end read data to produce a genome assembly of 173 Mb (designated *Eg*-G1s; scaffold N50/N90 = 18.7/12.3 Mb; scaffold L50/L90 = 4/8; Table 1). This assembly (*Eg*-G1s) comprised 9 scaffolds (512 contigs) that were consistent with chromosomes and represented 97% of the genome (Fig. 1), with <4.6 Mb of sequence present in 5 scaffolds (17 contigs). Contiguity of the *Eg*-G1s assembly (Table 1) was substantially greater than the 371 scaffolds (3736 contigs)^14^ and 957 scaffolds (8264 contigs)^16^ achieved for *E. granulosus* in previous studies. The assembly was ‘polished’ with short-reads (25.1 Gb with 167-fold coverage; Supplementary Table 1) from the same *E. granulosus* sample to correct indels and single nucleotide alterations, with 97% of these reads mapping back to the “polished” assembly (Supplementary Table 1).

**Table 1:** Genome features.

Characteristics	*Echinococcus granulosus* (Eg-G1s)	*Echinococcus granulosus*^a^	*Echinococcus granulosus*^a^	*Echinococcus multilocularis*^a^
Genome size (bp)	172,983,221	114,538,160	110,837,706	114,963,242
Number of scaffolds (contigs)	31 (542)	1288	957	1217
N50 (bp); L50 of contig assembly	1,386,608; 24	–	–	–
N90 (bp); L90 of contig assembly	114,310; 218	–	–	–
N50 (bp); L50 of scaffolded assembly	18,675,433; 4	5,228,736; 8	712,683; 39	13,762,452; 4
N90 (bp); L90 of scaffolded assembly	12,340,804; 8	213,489; 41	127,284; 181	2,924,275; 10
Genome GC content (%)	42.2	41.9	41.8	42.2
Repetitive sequences (%)	36.2	10.55	-	11.95
Exonic proportion; including introns (%)	9.0; 33.1	13.3; 48.8	14.3; 55.6	13.7; 49.1
Number of putative coding genes	9985	10,245	11,319	10,663
Mean; median gene size (bp)	5727; 2912	5459; 2692	5481; 3281	5292; 2654
Mean; median CDS length (bp)	1551; 1095	1486; 1062	1401; 939	1476; 1041
Mean exon number per gene	7.0	6.8	6.7	6.8
Mean; median exon length (bp)	221; 159	219; 159	211;153	218; 158
Mean; median intron length (bp)	693; 240	685; 247	722; 318	663; 242
Coding GC content (%)	50.1	50.0	49.3	49.9
BUSCO completeness: complete; partial genes (%)	69.9; 6.2	71.9; 5.5	69.2; 5.7	72.6; 5.2

Comparison of the characteristics of the genome *Eg*-G1s of *Echinococcus granulosus* (genotype G1) with those of previous draft genomes of *E. granulosus* (G1) and *E. multilocularis*.

^a^Short-read assemblies^14,15^.

### Long genomic repeat regions

Conspicuous in the *Eg*-G1s genome were five long repeat regions with low densities of genes on chromosomes 1, 2, 4, 7 and 8 (Fig. 1). Repeat regions in chromosome 1 harboured 23 protein-coding genes and ten 5 S ribosomal (r)RNA genes; the beginning of chromosome 2 had 7 genes coding for proteins including histones H2B/H3/H4, and 10 large subunit (*LSU*), small subunit (*SSU*), 5.8 S and 5 S rRNA, and U2 spliceosomal small non-coding (sn)RNA genes (Supplementary Data 1; Fig. 1a); the end of chromosome 2 had a long (~ 11.5 Mb) repetitive tract of *LSU*, *SSU*, 5.8 S rRNA and U2 spliceosomal snRNA genes (*n* = 44) and 21 protein-coding genes (Supplementary Data 1; Fig. 1a). Repeat regions were distributed across chromosome 4 and contained *LSU*, *SSU*, 5.8 S rRNA and U2 spliceosomal snRNA genes (*n* = 80) and 104 protein-coding genes including some encoding histones (H2A, H2B, H3 and H4) (Supplementary Data 1; Fig. 1a). Repeat regions at the end of chromosomes 7 and 8 contained 46 and 67 protein-coding genes, respectively, with mainly U3 spliceosomal snRNA genes (*n* = 8) uniquely present in chromosome 7 (Supplementary Data 1; Fig. 1a). A comparison of the nature and extent of repeat regions (Supplementary Tables 2 and 3; Supplementary Data 2 and 3) revealed that only chromosomes 1 and 2 have similarities in that they share 18 of the most frequent repeat elements with each other, with non-coding RNA gene regions being unique to the latter chromosome; all other chromosomes have distinctly different repeat and gene contents (Supplementary Table 3; Supplementary Data 1, 2, 4 and 5).

Long repetitive genomic regions cannot be assembled using short-read sequencing approaches (e.g., Illumina), which is why previous genome assemblies for *Echinococcus* species^14,16^—although likely comprising most protein-coding genes—were one-third smaller than the genome size inferred here (173 Mb; Table 1). In accord with recent studies^17–20^, we demonstrate here the advantage of using long-read and complementary sequencing methods to overcome fragmentation in the assembly of complex genomes. The proximity of the *Eg*95 genes to the long repetitive regions identified here raises a question as to whether these regions play roles in regulating the transcription and/or expression of these genes via non-coding RNAs, warranting future exploration.

### Gene content and comparison among species

To support gene predictions and explore transcription in key developmental stages of *E. granulosus* genotype G1, we produced RNA-seq data for both the adult worm and oncosphere stages and sourced publicly available data for the protoscolex stage^14,16^. All of these data were mapped and transferred to the assembled genome (*Eg*-G1s), and 9985 protein-coding genes were identified (Table 1). As this genome assembly exhibited features consistent with ‘reference quality’^21^, including high contiguity and N50/N90, very few gaps and unplaced sequences, and evidence of high-quality protein-coding genes (cf. Table 1), we defined this version as a chromosome-level reference genome (*Eg*-G1s; accession no. PRJNA754835 in NCBI) whose proteome-completeness metrics (BUSCO) were similar to those achieved previously for *E. granulosus* and *E. multilocularis*^14^ (Table 1). Having validated assembly-quality, we then assigned functions to 8972 (89.9%) of the 9985 protein-coding genes in the *Eg-*G1s genome (Supplementary Data 4); 1013 (10.1%) genes could not be annotated, 593 (5.9%) of which were transcribed (‘unknowns’ or orphans) and 208 (2.1%) of which encoded proteins with domains, motifs or signatures consistent with excretory/secretory or membrane-bound molecules (Supplementary Data 4).

**Fig. 2:**
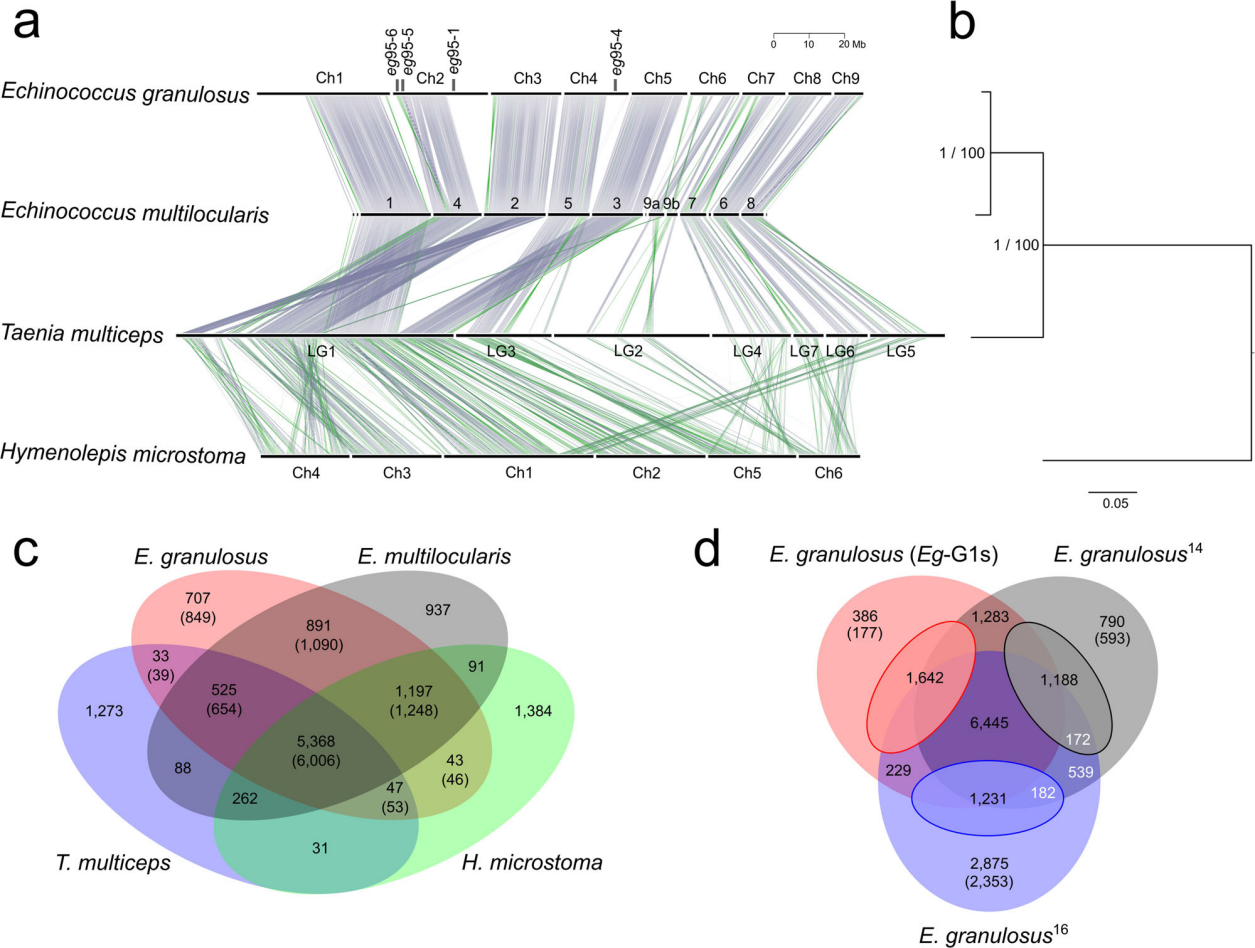
Synteny, relationships and orthology. **a** Synteny of the nine chromosomes (Ch1 to Ch9) of the genome *Eg-*G1s of *Echinococcus granulosus* (genotype G1) with scaffolds or chromosomes in the genome assemblies of *E. multilocularis*^14^, *Taenia multiceps*^22^ and *Hymenolepis microstoma*^23^. Each line represents a single copy orthologous (SCO) gene between two species (grey—same orientation; green—reverse orientation). Scale bar (top right) indicates chromosome length (Mb). **b** Consensus tree showing the genetic relationship of the four cestode species using data for 4040 shared SCOs (nodal support values: 1.0 and 100% for MrBayes and RAxML analyses, respectively; scale bar: substitutions per sequence site). **c** Venn diagram displaying the numbers of orthogroups between or among the four cestode species obtained using the program OrthoFinder^68^ (numbers of *E. granulosus* genes in parentheses). **d** Venn diagram comparing the numbers of genes (using OrthoFinder) common or distinct between or among the reference genome *Eg-*G1s (top left) and previously published assemblies^14,16^. Numbers of paralogous genes (small ovals) and orthologous and/or single copy genes (large ovals and overlaps) are indicated, as are orphan (unknown) genes (in parenthesis). Numbers of gene predicted (*n* = 1432; 539 + 172 and 539 + 182) from two previous draft genomes of *E. granulosus*^14,16^ for which homologous protein-coding genes were not identified in the final gene set of *Eg-*G1s. White lettering was used only to improve visibility of numbers on dark background.

In a comparative analyses, we identified more paralogous genes (*n* = 1642) in *Eg*-G1s than in previous draft assemblies for *E. granulosus* (1188 and 1231, respectively)^14,16^ (Fig. 2d), with ‘novel’ paralogs being discovered in long repeat regions containing gene families encoding proteins including histones H2A, H2B & H4 and variable surface glycoproteins (Supplementary Table 4), and we found fewer unique orphan (unknown) proteins encoded in *Eg*-G1s than in previous assemblies (Fig. 2d). Although the gene sets predicted for previous *E. granulosus* draft genomes^14,16^ contain 1432 protein-coding genes without homologues in *Eg*-G1s (Supplementary Data 6; Fig. 2d), 486 of them are orphans (Supplementary Data 6). However, before the removal of 652 low-confidence genes from *Eg*-G1s using stringent criteria (Supplementary Fig. 1; Supplementary Data 7), the differential gene set was 771 genes, including 370 orphans (Supplementary Data 6). Thus, the final *Eg*-G1s gene set (*n* = 9985) compares to that of *E. multilocularis*, with 8998 homologues shared with *Taenia multiceps* (*n* = 6611) and *Hymenolepis microstoma* (*n* = 7293) (Fig. 2c).

### Marked synteny between *E. granulosus* and other tapeworms

Pairwise comparisons revealed that there was notable synteny between the *Eg-*G1s and published genome assemblies for *E. granulosus*^14^, *E*. *multilocularis*^14^, *T. multiceps*^22^ and *H. microstoma*^23^ (Fig. 2; Supplementary Fig. 2). As expected, most synteny was observed between *Eg*-G1s and the *E. multilocularis* reference genome (Fig. 2; Supplementary Data 8), with 92.8% of nucleotides (*n* = 98,392,918 bp; 10 scaffolds) of the latter aligning to 72.9% of nucleotides (*n* = 125,154,915 bp; 11 scaffolds) of the former in 477 syntenic blocks. There was less synteny between *Eg*-G1s and each *T. multiceps* and *H. microstoma* (Fig. 2; Supplementary Data 8), with 42.2% and 61.1% of nucleotides in *T. multiceps* and *H. microstoma* aligning to 55.8% to 38.6% of nucleotides in the *Eg*-G1s genome (9 scaffolds) in 50 to 270 syntenic blocks, respectively (Supplementary Data 8). The observed syntenies (Fig. 2a) were compared with phylogenetic distances (Fig. 2b) between/among species.

### Chromosomal localisation of four *Eg*95 genes and their transcription

The high-quality *Eg*-G1s reference genome (Table 1; Fig. 1) provided the basis to localise members of the *Eg*95 gene family in the genome, as previous attempts had failed due to the fragmented nature of draft genomes assembled using short-read data sets alone^13^. Here, we defined four distinct genes, *Eg*95-1, *Eg*95-4, *Eg*95-5 and *Eg*95-6. Four of the previously-characterised alleles, *eg*95-1, *eg*95-4, *eg*95-5 and *eg*95-6^10^, were unequivocally assigned to genes *Eg*95-1, *Eg*95-4, *Eg*95-5 and *Eg*95-6, and the two other alleles *eg*95-2 and *eg*95-3^10^ could be assigned to *Eg*95-1 and/or *Eg*95-4 (but not unequivocally to either due to their substantial nucleotide sequence identity: 99.2%). Genes *Eg*95-1 (encoding protein EG95^10^) and *Eg*95-4 were localised to chromosomes 2 and 4, respectively, and genes *Eg*95-5 and *Eg*95-6 were at the end of chromosome 2 (Fig. 1). All four *Eg*95 genes are encoded in repeat-rich regions of the genome; *Eg*95-5 and *Eg*95-6 are close to one another and to the end of chromosome 2 (Fig. 1).

**Fig. 3:**
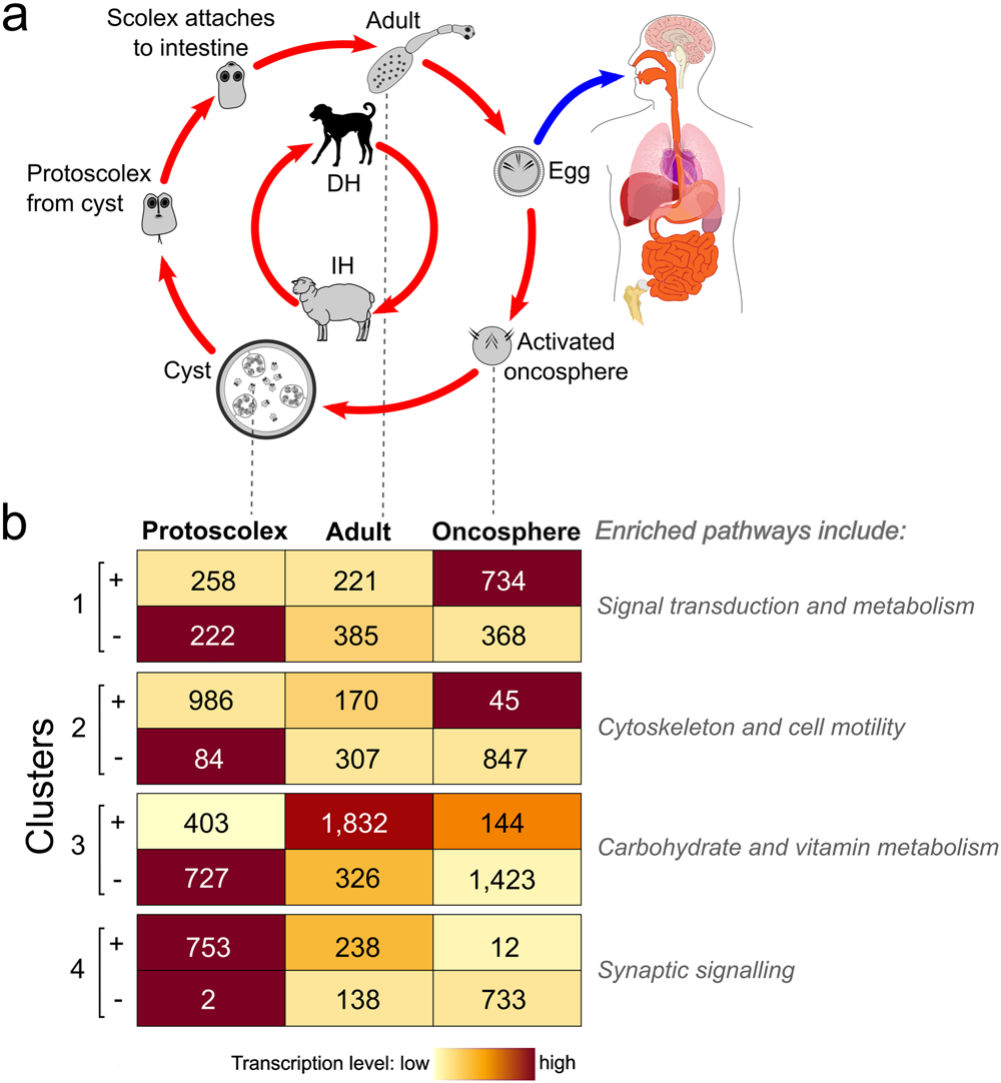
Transcription in *Echinococcus granulosus* (genotype G1). **a** Life cycle of *E. granulosus* with key developmental stages indicated – adapted from ref. ^23^ – canid definitive host (DH); intermediate host (IH). **b** Four distinct clusters (each with sub-clusters + and –; divided according to fold-change (FC) ≥ 4 and FC ≤ −4, respectively) of genes whose transcription correlated among the protoscolex, adult and oncosphere stages, inferred by weighted correlation network analysis (numbers in boxes are gene counts). The four *Eg*95 genes (within sub-cluster 1 + ) are highly transcribed in the oncospheral stage. Enriched biological (KEGG) pathways representing individual gene clusters/sub-clusters are indicated. White lettering was used only to improve visibility of numbers on dark red background.

Exploring transcription in the distinct developmental stages of *E. granulosus* gave insight into biological processes and pathways. Genes *Eg*95-1 (log_2_ FC = 15) and *Eg*95-4 (log_2_ FC = 12) had the highest transcription in the activated oncosphere stage, followed by *Eg*95-5 (log_2_ FC = 7) and *Eg*95-6 (log_2_ FC = 6), with reference to the protoscolex stage, whereas all four *Eg*95 genes had low levels of transcription in adult worms of *E. granulosus* (Supplementary Table 5; Supplementary Data 6; Supplementary Fig. 3). A weighted network analysis defined four distinct clusters (each with sub-clusters ‘+’ and ‘–’) of genes whose transcription was correlated among the protoscolex, adult and oncosphere stages. In the activated oncosphere, the 915 genes that grouped with the four *Eg*95 family members (all within cluster 1; Fig. 3) were inferred to be linked to key biological pathways, including genetic information processing (ribosome/translation; folding, sorting and degradation/proteasome; protein processing in endoplasmic reticulum; DNA replication and repair); environmental information processing (signal transduction and signalling molecules; Rap1, Ras, PI3K-Akt, Notch and JAK-STAT); cellular processes (focus adhesion and adherens junction); and metabolism (amino acid and energy) (Fig. 3; Supplementary Data 9 and 10). Other clusters of genes were associated with environmental information processing (signalling), organismal systems (endocrine) and cellular processes (e.g., cell motility and cytoskeleton) (cluster 2); metabolism (carbohydrate, vitamin and co-factors), organismal systems (e.g., carbohydrate digestion/absorption, endocrine and excretion/absorption) (cluster 3) or environmental information processing (signal transduction), cellular processes (e.g., regulating pluripotent stem-cells) and organismal systems (nervous/synapse, endocrine and development/regeneration) (cluster 4) (Fig. 3; Supplementary Data 9 and 10). While *Eg*95 genes do not map to currently-known biological pathways or processes, their high transcription in the oncosphere associates with at least 1102 other cluster 1-genes linked to extensive cellular signalling, metabolism and adhesion, in accord with essential processes required for the parasite to invade/infect the intermediate host animal, including the penetration of the small intestinal wall using oncospheral hooks and excretions/secretions from the penetration glands—in which EG95 is expressed^12^—to then enter lacteals and/or capillaries for subsequent passive transport to the liver and/or lung, where this stage undergoes post-oncospheral alteration to initiate cyst development (Fig. 3).

### Conservation of EG95-1 and related molecules

**Fig. 4:**
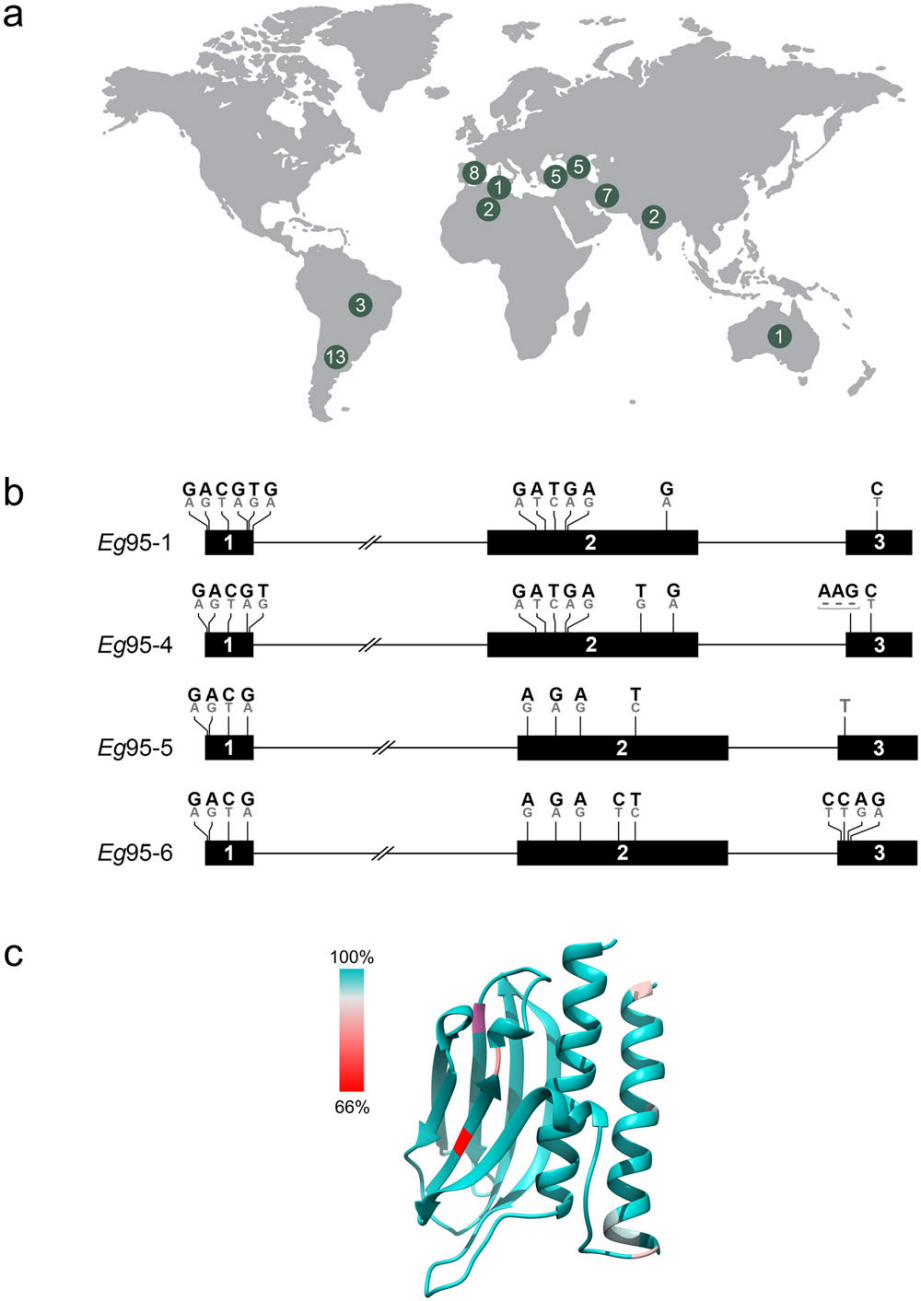
Assessment of genetic variation in the *Eg*95-1 gene and associated gene product. **a** Genomic DNA samples (*n* = 47) representing single cysts or adult worms of *Echinococcus granulosus* (genotype G1 or G3) from 8 distinct host species and 10 different countries were sequenced. **b** Mapping of sequence data from individual samples to the haploid reference genome (*Eg-*G1s) sequence detected polymorphism (allelic variability) but no unambiguous (i.e. fixed or homozygous) nucleotide difference in the 3 exons of *Eg*95-1 for any of the (diploid) sequences from any of the 47 samples with the reference sequence. Black horizontal bars represent the three exons (1 to 3) and black lines denote intervening introns. Polymorphic positions are indicated above each exon: a dominant base (black) matches the *Eg-*G1s reference sequence; a grey base represents the minor allele (cf. Supplementary Data 13); a fixed nucleotide difference from the reference sequence is indicated at one position; and a dash indicates an indel. **c** Mapping of allelic variation of EG95-1 to the modelled three-dimensional structure of the vaccine molecule EG95 reveals variable regions (see colour-key for percentage conservation) in the N-terminal α-helix, as well as two β-strands, each of which located in one of the predicted anti-parallel β-sheets. All residue side chains subject to allelic variation are surface exposed, and thus, due to the conservative nature of most mutations (A→T, T→I, G→E, M→R, V→I, R→H, E→D, D→S), overall structural conservation of the vaccine molecule (EG95-1) can be inferred.

Studies conducted in a range of countries, including Australia, Argentina and China, have shown that the recombinant EG95 vaccine consistently induces high levels (95–99%) of protection in the intermediate host (sheep) against challenge infection with *E. granulosus* eggs^6,7^. However, no study has yet comprehensively assessed sequence variation in *Eg*95-1 and related genes within *E. granulosus* in relation to geographical and/or host origin. To explore sequence variation in the gene encoding EG95-1, high-quality short-read genomic data (mean: 31 Gb) for each of 47 *E. granulosus* samples were mapped (coverage: 100%; depth at each nucleotide position: ≥20; mean depth: >80) to the *Eg*95-1 gene within the genome (Supplementary Data 11). No fixed nucleotide difference was detected (upon pairwise comparison) in the open reading frame (ORF = 3 exons) of this gene for any of the 47 individual samples when compared with the reference genome sequence (*Eg*-G1s) (Fig. 4). Although minor polymorphism (allelic variability) was detected at 13 positions in all 3 exonic regions of *Eg*95-1 (Fig. 4; Supplementary Data 12), the dominant base at each of these positions matched the reference sequence (Supplementary Data 13). This heterozygosity, detected also in PacBio long-read and transcriptomic data sets, was expected because *Eg*-G1s is presented as a haploid reference representing a diploid organism (i.e., *E. granulosus*^24^).

The amino acid conservation inferred here for the vaccine molecule (i.e. EG95-1), based on the mapping of short-read data derived from *E. granulosus* (G1 or G3) from sheep or other host species, including cattle, buffalo, goat, pig, dog/dingo and human from 10 different countries (Fig. 4; Supplementary Data 11) is consistent with the biological evidence of a consistently high degree of protection achieved by the EG95 vaccine in sheep against cystic echinococcosis, irrespective of geographical location^6,7^, and also in accord with findings from some previous investigations indicating molecular conservation of EG95-1 in *E. granulosus* genotype G1^25,26^. The evidence of conservation in EG95-1 contrasts the results from one study^27^, suggesting marked nucleotide variability in *Eg*95-1, which we interpret might relate to artefacts introduced due to the methodology employed at the time; the PCR-primers employed match both genes *Eg*95-1 and *Eg*95-4 and, thus, would have co-amplified these and potentially other genes and/or might have created artefactual ‘chimeras’ in PCR, ultimately being reflected in apparently variable sequences ensuing molecular cloning and sequencing.

Our findings, using both long-read and short-read data, provide clear evidence of heterozygosity in *Eg*95-1 (Fig. 4) and in the three other *Eg*95 genes (Supplementary Data 12). At this point, we are not able to conclude whether this allelic variation is present within cells, among distinct cell types within individual developmental stages of *E. granulosus*^12^ or among individuals within cysts or worm populations (i.e. samples), but we speculate that this allelic variation for members of the *Eg*95 gene family is critical for adaptation to distinct host species and survival. Major transcription of *Eg*95-1 in activated oncospheres, but not in adult worms (containing intact eggs), is consistent with immunohistochemical evidence of pronounced expression of the EG95 protein in penetration glands within activated, infective oncospheres, but substantially less in eggs^12^. Interestingly, as EG95 appears not to be expressed on the tegument of the activated oncosphere stage, it is proposed that this infective stage is killed by complement-mediated antibody attack in EG95-vaccinated sheep during early post-oncospheral development in tissues in lung or liver^12^.

## Conclusion

In addition to defining the genomic locations, structures and compositions of the *Eg*95 gene family members and demonstrating the conservation of the EG95-vaccine molecule, the genomic and transcriptomic resources created here pave the way for myriad future molecular explorations of cystic echinococcosis/*E. granulosus*. Further work will be required to comprehensively explore developmentally regulated gene transcription and expression using at least four replicate samples for individual stages of *E. granulosus*. Investigating the structural and functional roles of the long stretches of non-coding DNA in the genome would also be interesting. At the host-parasite interface, it would be exciting to explore the molecular ‘cross-talk’ between oncosphere and the immune system, and the function(s) of EG95 as a fibronectin III domain-containing molecule, possibly involving the use of well-defined liver and/or lung organoid systems^28,29^. On a broader scale, exploring molecular variation within *E. granulosus sensu stricto* from the host and distributional ranges across the globe, in genome-wide manner, could comprehensively document the population genetic sub-structuring, with implications for understanding transmission patterns of cystic echinococcosis. From an applied perspective, the inference and functional evaluation of essential genes encoded in the *Eg-*G1s genome could enable the discovery of new intervention targets for the treatment of cystic echinococcosis in people. These are just some of the areas that should be positively impacted by the availability of a chromosome-scale genome and associated data and tools.

## Methods

### Ethics statement

No ethics permissions were required for this study. Samples of *E. granulosus* (cf. Supplementary Data 11) were collected from animals by logistical support personnel and professionals, with approval from relevant institutions in individual countries; samples were donated to the investigators of this article.

### Genomic sequencing

High molecular weight genomic DNA (1 µg) was isolated from protoscoleces (200 µl packed volume) from an individual cyst of *E. granulosus* (genotype G1) obtained from a sheep (*Ovis aries*) from New South Wales, Australia, using an established sodium dodecyl-sulphate–proteinase K digestion protocol and phenol/chloroform extraction^10^. The DNA amount was determined using a Qubit fluorometer dsDNA HS Kit (Invitrogen), and DNA integrity was verified using a Bioanalyzer 2100 (Agilent). For long-read sequencing, a SMRTbell library was constructed from 8 μg of genomic DNA (≥10 kb) without prior shearing, employing the SMRTbell Template Prep Kit 1.0, following the manufacturer’s protocol, and enriching for templates of >10 kb using the BluePippin system (Sage Scientific, MA, USA). This library was sequenced (chemistry v.2.1) in three SMRT cells using the PacBio Sequel System (Pacific Biosciences, Menlo Park, CA, USA). For short-read sequencing, a paired-end library (insert size: 500 bp) was constructed from 1 μg of genomic DNA using the MGIEasy DNA Library Prep Kit (V1.1, MGI Tech Co., Ltd, Shenzhen, China), employing the recommended protocol, and then sequenced (PE100 chemistry) using the BGISEQ-500RS platform.

### RNA-seq and transcription analysis

For the protoscolex stage of *E. granulosus* (genotype G1), RNA-seq data (8 samples) were obtained from the NCBI Sequence Read Archive (SRA; accession number SRP172517^30^). For adults and activated oncospheres^12^, one sample each, of *E. granulosus* (genotype G1), total RNAs were isolated from hundreds of individuals using the TriPure isolation reagent (Roche Molecular Biochemicals). RNA yield was estimated spectrophotometrically (NanoDrop 1000), and integrity verified using the BioAnalyzer (Agilent). RNA-seq was conducted using an established method^31^ on a NovaSeq 6000 instrument and relevant data summarized (cf. Supplementary Table 1). The genome-guided assembly of RNA-seq data was performed using a software pipeline, incorporating the program Trimmomatic v0.36^32^ for read quality filtering, Hisat2 v2.1.0^33^ for read mapping, Trinity v2.8.4^34^ for sequence assembly and CD-HIT-EST v4.81^35^ for reducing redundancy. EdgeR v3.32^36^ was used to estimate log_2_-fold change (FC) in transcription of individual *Eg*95 genes between each the adult or oncosphere and the protoscolex stage of *E. granulosus* employing an established protocol^37^ and using a minimum counts-per-million threshold of 0.35. For EdgeR, expected read-counts were calculated using the program RSEM v1.3.3^38^.

### Genome assembly

An established pipeline^21^ was used to create an initial assembly from PacBio sequence data. In brief, these data were assembled using the program Canu v1.8^39^, polished with both PacBio raw reads using the program Arrow^40^ and with BGISEQ-500 PE reads employing the software Pilon v1.22^41^. Redundant sequences were removed from the assembly using the program Purge Haplotigs v1.1.1^42^, and resultant contigs were combined with longer contigs using a customised workflow v0.0.1-publication (https://gitlab.unimelb.edu.au/vetscience/gapmaster), which includes the program RagTag v1.1.0 (https://github.com/malonge/RagTag) for scaffolding and TGS-GapCloser v1.0.3^43^ to close gaps. This workflow was run in an iterative manner, guided by previously published genome sequences for *E. granulosus*^14^ and *E. multilocularis*^14^, using high-quality, corrected long reads. The quality of gap closure was verified in each iteration; if there was any indication of a break point in the flanking regions of closed gaps, or if they had a repeat content of >50%, scaffolds were broken again into contigs. The process was repeated until no more gaps could be closed. Using Pilon, resultant combined contigs were iteratively polished both with short-read data to remove mismatches and indels, and with RNA-seq data (SRR8281957–SRR8281959, SRR8284434–SRR8284436 and SRR8293717–SRR8293719; for *E. granulosus* G1) to remove indels of <10 bp in length. Using RagTag, final scaffolding (Supplementary Data 14) was carried out using homologous sequences (without closing gaps) in genomes of *E. multilocularis* and *E. granulosus*.

### Gene prediction and functional annotation

Gene models were predicted using a custom pipeline (v0.0.1-publication; https://gitlab.unimelb.edu.au/bioscience/annotosis), which employs the programs AUGUSTUS v3.4.0^44^, StringTie v2.1.4^45^, GMAP v2020.10.14^46^, EMBOSS v6.6.0^47^, TransDecoder v5.5.0^34^ and CD-HIT 4.8.1^35^ using the same RNA-seq data as evidence utilised for gene and genome polishing as well as gene models from a previous *E. granulosus* assembly^14^ using the program LiftOver^48^ and all Swiss-Prot protein sequences within UniProtKB^49^ (accessed 15 March 2021). The quality of the predicted genes was assessed using a custom pipeline (v0.0.1-publication; https://gitlab.unimelb.edu.au/bioscience/annotosis), which builds on the programs Kmeans in the R language^50^, fLPS^51^, table2asn^52^, InterPro 5.51^53^ (https://www.ebi.ac.uk/interpro/), bedtools^54^, OrthoMCL v2.0.4^55^, GeneValidator v2.1.10^56^ and BUSCO 5.1.2^57^. Genes inferred to be of a low quality, based on the observed ‘steepest curvature before the shoulder point’ in the graph displaying the estimated gene-wise quality scores, were removed. The annotation of each inferred amino acid sequence was achieved using InterPro and sequence homology to proteins in the Swiss-Prot, KEGG^58^; accessed (30 June 2021) and NCBI NR^59^; accessed (4 February 2021) databases using BLASTp (threshold E-value: ≤10^−8^). Genes that were transcribed at ≥ 0.35 counts-per-million but not annotatable were designated as ‘unknowns’ or ‘orphans’. Nuclear *LSU*, *SSU*, 5.8 S and 5 S rRNA, spliceosomal snRNA were predicted by applying the program Infernal v1.1.4^60^ with Rfam 14 database^61^ to the assembly.

### Prediction of repeat regions

Genomic repeat elements specific to *E. granulosus* were first inferred using the programs RECON^62^ and RepeatScout^63^. These repeats were processed using the program RepeatModeler^64^ to obtain custom repeats, which were then combined with known repeats from Repbase v.17.02^65^ to mask the *Eg*-G1s assembly employing the program RepeatMasker^66^.

### Assessing genome completeness and synteny

First, the completeness of the *E. granulosus* genome (*Eg-*G1s) was assessed using the program BUSCO v5.1.2 (lineage: Metazoa). Second, the synteny of *Eg-*G1s with the published (repeat-masked) genomes of *E. granulosus*, *E. multilocularis*^14^, *T. multiceps* and *H. microstoma* was visually assessed using the program circos v.0.23^67^ by identifying genomic locations of protein-encoding single-copy orthologs (SCOs) in a pairwise manner and inferred using OrthoMCL v2.0.4^55^. Homologous genes between/among these species and previous *E. granulosus* assemblies^14,16^ were inferred using the program OrthoFinder v2.5.4^68^, and the numbers of shared homologous gene groups displayed in Venn diagrams. The numbers of orphan genes in previous *E. granulosus* assemblies^14,16^ were established via BioMart in WormBase ParaSite^69^.

### Phylogenetic analysis

Aligned amino acid sequences of SCOs among *E. granulosus* (genotype G1) genome, *E. multilocularis*, *T. multiceps* and *H. microstoma* were subjected to (unrooted) phylogenetic analyses using Bayesian inference (BI) in MrBayes v.3.2.2^70,71^ and maximum likelihood (ML) in RAxML v.8.0.24^72^. The evolutionary models were established using the program PartitionFinder v.2.1.1^73^. The number of Markov Chain Monte Carlo (MCMC)^74^ iterations for BI was 10 million generations, from which the first 25% were discarded as non-converged ‘burn-in’. For ML, nodal support values were assessed by 1000 bootstrap replicates. The resultant trees were then subjected to analysis in the program SumTrees using DendroPy v.3.12.0^75^ to produce a consensus tree, and drawn using the program FigTree v.1.4 (https://www.softpedia.com/get/Science-CAD/FigTree-AR.shtml).

### Network analysis, and pathway and functional enrichment

Using the program WGCNA v1.69, we performed a weighted correlation network analysis^76^ to define correlated clusters (modules) of genes in the protoscolex, adult and oncosphere stages of *E. granulosus* according to level of transcription (in TMM normalised expected counts) using a minimum cluster size of 500 genes and a scale-free index of 0.6. Qualitative transcription analysis was undertaken, as single adult and oncosphere samples were available (such samples are challenging to source and infective to humans). For the individual developmental stages, the clusters obtained were subdivided according to the log_2_-fold change (FC): ≥ 2 (high; h) or ≤ −2 (low; l). FCs were compared among the stages using the program egdeR v3.32.0^36^, in accord with best practice for qualitative analysis^37^. Expected read-counts used in WGCNA and in EdgeR, and TPMs used in a heatmap were calculated using the program RSEM. The enrichment of the Kyoto Encyclopaedia of Genes and Genomes (KEGG) pathways and the BRITE functional hierarchies^58^ was inferred using a well-established methodology^77^. For heatmap display, TPM values were averaged for the protoscolex stage.

### Short-read sequencing of genomic DNA samples from distinct host species and geographical locations around the world

Genomic DNA samples representing *E. granulosus* genotypes G1 (*n* = 41) and G3 (*n* = 6) (Supplementary Data 11) were available from previous studies^78–81^. Total genomic DNA had been extracted from protoscoleces or germinal membrane from single cysts from intermediate hosts species, or single adult worms of *E. granulosus* from individual canids, using the High Pure PCR Template Preparation Kit (Roche Diagnostics, Mannheim, Germany). DNA amounts were determined using a Qubit fluorometer dsDNA HS kit (Invitrogen). Then, individual DNA samples were whole genome-amplified using the REPLI-g Mini Kit (QIAGEN; cat. no. 150025), and genomic DNA libraries constructed using the MGIEasy FS DNA Library Prep Set (MGI; v2.0) and an established protocol^82^. The libraries were then sequenced (100 bp paired-end reads) using the DNBSEQ-T1 platform (BGI–Shenzhen, China).

### Recording nucleotide variation

For individual samples, raw DNA sequence data in the FASTQ format^83^ were filtered for quality using SOAPnuke v1.5.6^84^ by removing adapter-contaminated, duplicated and low-quality reads (parameters -l 20, -q 0.3, -n 0.02 and -d). Sequence quality was verified using FastQC v0.11.8^85^ and MultiQC v1.7^86^. Then, high-quality read-pairs were mapped to the nuclear genome sequence of *E. granulosus* (*Eg*-G1s) using the Burrows-Wheeler Aligner (BWA) v.0.7.8^87^ and kept in the BAM format. Subsequently, read coverage, depth and mapping quality scores in all four *Eg*95 genes were assessed for each individual sample using mosdepth v.0.3.1^88^. For each sample, the aligned read data was then used to record single nucleotide polymorphisms (SNPs) at individual positions and insertion/deletion events (indels) in relation to the reference genome sequence using the Genome Analysis Toolkit (GATK) v4.1.3.0^89^. In brief, base quality scores of ‘raw’, aligned read data were re-calibrated twice based on predicted variants; then, SNP sites and indels were identified for each sample using the GATK HaplotypeCaller^89^ and merged into one ‘variant call format’ (VCF) file – listing all variable sites for all samples – using GATK Combine GVCFs and GenotypeGVCFs. ‘Raw’ SNPs and indels were filtered for quality using GATK VariantFiltration, retaining SNPs if strand bias (FS) < 60, variant confidence (QD) > 1.0, mapping quality (MQ) > 20.0, mapping quality (MQRankSum) > −12.5, read position bias (ReadPosRankSum) > −8.0, and indels if FS < 200, QD > 2.0, ReadPosRankSum > −20.0. Variable sites were verified by eye using the read alignment file and the program Geneious v.11.1.5^90^.

### Analyses of nucleotide variation for individual *Eg*95 genes

For each of the 47 *E. granulosus* DNA samples, individual FASTA files containing sequence data (including inferred SNPs and indels) for each gene were generated using GATK FastaAlternateReferenceMaker and BCFtools v1.9^91^, with all polymorphic substitutions coded as IUPAC ambiguity characters^92^. Subsequently, all gene sequences produced here were aligned, as were their amino acid sequences. The open reading frame (ORF) of each gene was verified, conceptually translated, and synonymous or non-synonymous substitutions identified using the program Geneious. All nucleotide sequences were deposited in the GenBank database (accession nos. MZ889937–MZ890124). The three-dimensional structure of EG95-1, conceptually translated from individual nucleotide sequences, was modelled using a deep learning method, employing RoseTTAFold software^93^ accessed via the protein structure prediction service Robetta (https://robetta.bakerlab.org/) that is continually evaluated through CAMEO (https://www.cameo3d.org/). Visualisation and figure preparation were done with UCSF Chimera (http://preview.cgl.ucsf.edu/chimera/).

### Reporting summary

Further information on research design is available in the Nature Research Reporting Summary linked to this article.

## Supplementary information

Supplementary Information (new)

Description of Additional Supplementary Files

Supplementary Data 1-14

Reporting Summary

Supplementary Information

## Data Availability

The nucleotide sequence data from this study are publicly available via the NCBI database: BioProject PRJNA754835 (all genomic and transcriptomic data sets relating to genome *Eg*-G1s); GenBank accession no. JAIKUZ000000000 (*Eg*-G1s genome sequence); Sequence Read Archive (SRA) accession nos. SRR15522570, SRR15522571 and SRR15522580 (PacBio long read DNA data for the protoscolex stage of *E. granulosus* genotype G1); SRR15522572 to SRR15522577, SRR15522581 and SRR15522582 (short-read DNA data for the protoscolex stage of *E. granulosus* genotype G1); SRR15522578 (RNA-seq data for the oncosphere stage of *E. granulosus* genotype G1); SRR15522579 (RNA-seq data for the adult stage of *E. granulosus* genotype G1). GenBank accession nos. MZ889937 to MZ890124 (DNA sequences of each of the four *Eg*95 genes of each of 47 *E. granulosus* samples (genotype G1 or G3; derived from short read data)).
