# Supplementary material for: Chromosome-scale Echinococcus granulosus (genotype G1) genome reveals the Eg95 gene family and conservation of the EG95-vaccine molecule
Source: Commun Biol. 2022 Mar 3;5:199. doi: 10.1038/s42003-022-03125-1 (PMC8894454; doi:10.1038/s42003-022-03125-1)
Supplement: Supplementary file 1 — Supplementary Information (new) [file 42003_2022_3125_MOESM1_ESM.pdf]

Supplementary Table 1 | Sequence read data. Statistics for the *Echinococcus granulosus* genome (*Eg*-G1s) read data

Read library	Nucleotide count (bp)	Genome coverage (times)	Number of reads
PacBio reads	31,741,108,230	212	5,247,273
Illumina 250 bp insert paired end reads	25,128,153,000	167	251,281,530
RNA-seq reads for Oncospheres	14,351,109,600	NA	143,511,096
RNA-seq reads for Adults	20,807,757,900	NA	208,077,579

Supplementary Table 2 | Repeat content. Repeat content of the *Echinococcus granulosus* genome (*Eg*-G1s)

Element	Count	Length occupied (bp)	Percentage of genomic sequence (%)
Total interspersed repeats	-	61,416,555	35.50
SINEs	341	36,959	0.02
LINEs	2,920	169,593	0.10
LINE1	848	52,499	0.03
LINE2	454	24,600	0.01
L3/CR1	227	13,050	0.01
LTR elements	8,247	738,603	0.43
ERLV	31	1,597	0.00
ERLV-MaLRs	5	196	0.00
ERV class I	479	24,997	0.01
ERV class II	265	13,293	0.01
DNA elements	5,550	346,524	0.20
hAT-Charlie	173	10,441	0.01
TcMar-Tigger	35	2,224	0.00
Unclassified	94,877	60,124,876	34.76
Small RNA	2,133	303,239	0.18
Satellites	709	56,279	0.03
Simple repeats	27,518	1,271,317	0.73
Low complexity	3,139	147,206	0.09
Total bases masked		62,662,790	36.22

Supplementary Table 3 | Frequent repeat elements. Occurrences of the 20 most frequent repeat elements in the large repeat regions of the *Echinococcus granulosus* genome (*Eg*-G1s)

Repeat element	Chromosome 1 > 24,500,000 bp	Chromosome 2 > 15,900,000 bp	Chromosome 4 > 12,500,000 bp	Chromosome 7 > 9,400,000 bp	Chromosome 8 > 8,400,000 bp
DF0000652	0	0	0	0	62
GA-rich	0	168	0	52	95
rnd-3_family-103	432	267	0	0	0
rnd-3_family-11	0	0	0	0	1335
rnd-3_family-165	0	0	389	0	477
rnd-3_family-167	1087	847	0	0	0
rnd-3_family-192	705	400	0	0	0
rnd-3_family-193	457	301	0	0	0
rnd-3_family-202	1004	645	114	0	0
rnd-3_family-34	0	0	0	162	0
rnd-3_family-35	0	0	0	171	0
rnd-3_family-53	0	0	0	211	0
rnd-3_family-762	0	0	93	0	0
rnd-3_family-83	0	0	0	0	900
rnd-3_family-88	0	0	102	0	0
rnd-4_family-1125	0	0	83	0	0
rnd-4_family-116	0	0	155	0	0
rnd-4_family-1253	0	0	0	0	63
rnd-4_family-131	0	0	0	89	0
rnd-4_family-133	0	0	128	52	0
rnd-4_family-1591	0	0	0	0	53
rnd-4_family-17	556	264	0	0	0
rnd-4_family-18	390	231	0	0	0
rnd-4_family-239	0	0	0	0	131
rnd-4_family-242	0	0	0	0	86
rnd-4_family-24	340	198	0	0	0
rnd-4_family-266	0	0	108	0	77
rnd-4_family-267	0	0	158	0	0
rnd-4_family-349	200	145	0	0	0
rnd-4_family-379	0	261	499	0	0
rnd-4_family-383	0	0	210	0	183
rnd-4_family-384	0	0	135	0	131
rnd-4_family-421	0	0	0	0	119
rnd-4_family-43	0	0	95	0	0
rnd-4_family-469	0	0	85	0	0
rnd-4_family-470	0	0	162	0	0
rnd-4_family-49	0	0	0	189	0
rnd-4_family-616	0	187	0	0	54
rnd-4_family-723	369	201	0	0	0
rnd-4_family-765	0	0	0	127	0

rnd-4_family-770	855	638	0	0	0
rnd-4_family-858	411	262	0	0	0
rnd-4_family-971	0	0	150	0	0
rnd-4_family-97	383	223	0	0	0
rnd-5_family-12	249	197	0	0	90
rnd-5_family-1239	326	221	0	0	0
rnd-5_family-13	444	339	0	0	0
rnd-5_family-1394	0	0	0	0	71
rnd-5_family-1395	0	0	0	0	119
rnd-5_family-1521	0	0	0	0	91
rnd-5_family-2109	201	0	0	0	0
rnd-5_family-2153	0	0	0	99	0
rnd-5_family-217	0	0	100	0	0
rnd-5_family-221	0	0	0	81	0
rnd-5_family-223	0	0	0	81	0
rnd-5_family-2438	0	0	0	57	0
rnd-5_family-245	0	0	0	0	95
rnd-5_family-2676	0	0	0	0	53
rnd-5_family-389	0	0	0	51	0
rnd-5_family-4054	0	0	0	53	0
rnd-5_family-593	294	271	239	87	0
rnd-5_family-633	0	0	110	0	0
rnd-5_family-650	0	0	0	54	0
rnd-5_family-716	0	0	0	100	0
rnd-5_family-718	0	0	0	75	0
rnd-5_family-815	253	0	0	0	0
rnd-5_family-853	0	0	0	54	0
rnd-5_family-962	0	0	0	116	0

Supplementary Table 4 | Gene copy numbers. Differences in the copy number (n ≥ 5) of the predicted genes among three *Echinococcus granulosus* genomes (i.e. *Eg*-G1s reference and two draft genomes published previously[1,2]

Orthogroup ID	Gene count in *Eg*-G1	Gene count in *Eg*[a]	Gene count in *Eg*[b]	Total gene count in orthogroup	Description
OG0000000	69	2	3	74	Histone H2A[c]
OG0000001	68	2	2	72	Histone H2B[c]
OG0000002	59	0	0	59	Variant Surface Glycoprotein[c]
OG0000003	33	0	2	35	Histone H4[c]
OG0000004	19	1	1	21	Histone H2A[c]
OG0000005	16	1	0	17	Hypothetical protein[d]
OG0000006	less	1	0	17	Inosine triphosphate pyrophosphatase[c]
OG0000007	0	5	11	16	Retrovirus-related Pol polyprotein[e]
OG0000008	10	3	2	15	RNA helicase
OG0000009	13	0	0	13	Origin recognition complex subunit[c]
OG0000010	11	0	1	12	Mucin-22 like protein[c]
OG0000011	9	1	1	11	Hypothetical protein[c]
OG0000012	6	3	1	10	Multidrug andc toxin extrusion protein[c]
OG0000013	8	1	1	10	Inosine triphosphate pyrophosphatase[c]
OG0000014	6	1	3	10	Hypothetical protein[f]
OG0000016	7	1	1	9	Ureohydrolase
OG0000019	9	0	0	9	Origin recognition complex subunit[f]
OG0000020	8	1	0	9	Hypothetical protein[f]
OG0000022	0	2	6	8	Hypothetical protein
OG0000023	0	3	5	8	Retrotransposon-derived protein[e]
OG0000026	6	1	1	8	Hypothetical protein[c]
OG0000027	6	1	1	8	Hypothetical protein
OG0000029	5	3	0	8	Cysteine protease
OG0000030	0	0	7	7	Hypothetical protein
OG0000037	0	5	2	7	Heat shock protein
OG0000047	7	0	0	7	Rho GTPase activation protein
OG0000049	0	1	5	6	Retrovirus-related Pol polyprotein[e]
OG0000051	0	1	5	6	Hypothetical protein
OG0000058	0	0	6	6	Hypothetical protein
OG0000061	0	5	1	6	Hypothetical protein
OG0000066	5	0	1	6	DNA replication licensing factor
OG0000080	6	0	0	6	Neurofilament heavy polypeptide
OG0000081	6	0	0	6	Phosphotidylinositol phosphatase
OG0000082	5	1	0	6	Hypothetical protein
OG0000089	0	0	5	5	Hypothetical protein
OG0000237	5	0	0	5	Hypothetical protein[f]

[a]Short read *Echinococcus granulosus* assembly[1]
[b]Short read *Echinococcus granulosus* assembly[2]
[c]Located mainly in repeat regions of *Eg*-G1s
[d]Homologous to EmuJ_000357400 in *Echinococcus multilocularis*

[e]Putative transposons
[f]Located partially in repeat regions of *Eg*-G1s

Supplementary Table 5 | *Eg*-95 transcription. Differential transcription of Eg95 genes in *Echinococcus granulosus* genome (*Eg*-G1s)

Gene annotation	Gene ID	log_2 FC
Oncospheres vs. protoscoleces		
EG95-1	ECG_03412	15.55
EG95-4	ECG_06032	12.2756
EG95-5	ECG_02114	6.58807
EG95-6	ECG_02112	5.48484
Adults vs. protoscoleces		
EG95-1	ECG_03412	15.55
EG95-4	ECG_06032	12.2756
EG95-5	ECG_02114	6.58807
EG95-6	ECG_02112	5.48484

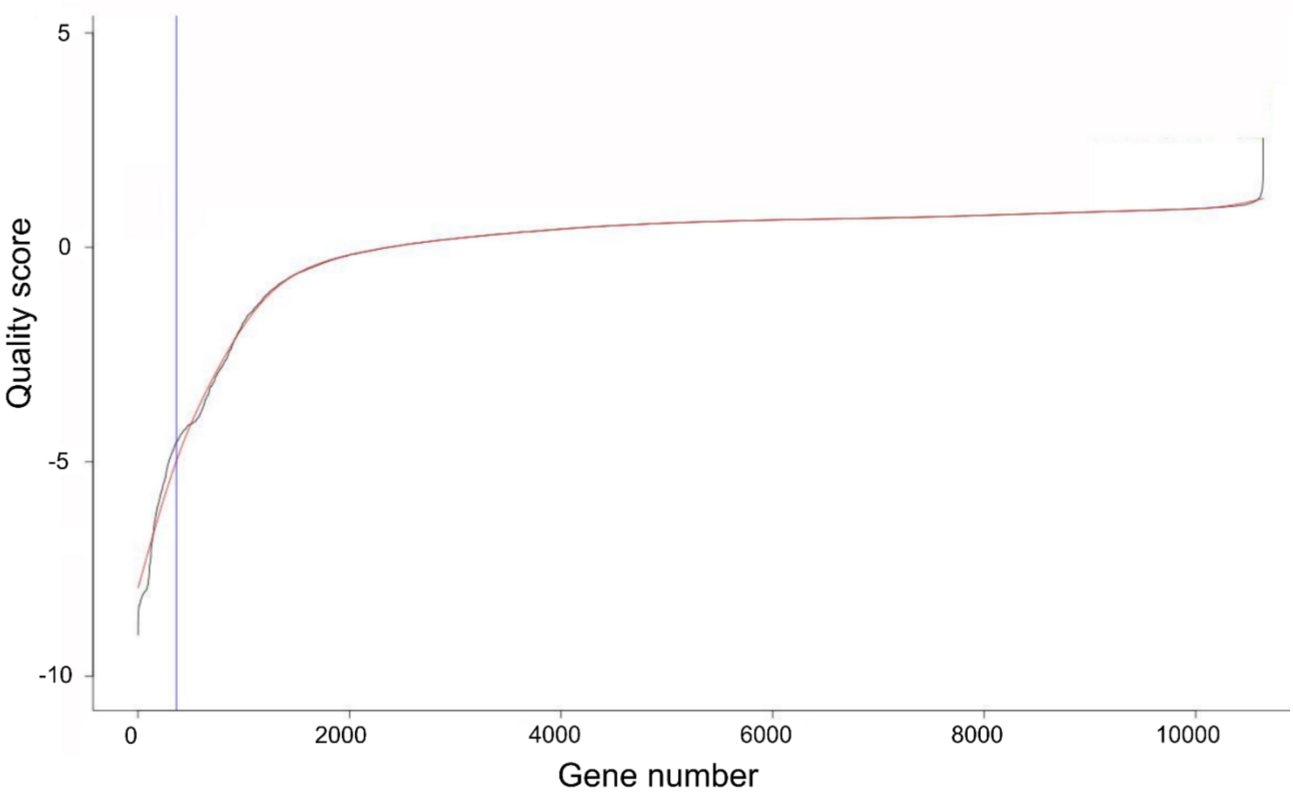

Supplementary Fig. 1. Scoring the quality of predicted genes. The graph displays the quality scores for 10,638 genes, in which a higher quality score reflects a more confident gene prediction. A black curve displays the calculated quality scores for individual genes. The smoothened red curve was used to estimate the cut-off (blue vertical line) for a low quality gene prediction (left of the line). The cut-off was set at the steepest curvature value of the red curve before the shoulder point.

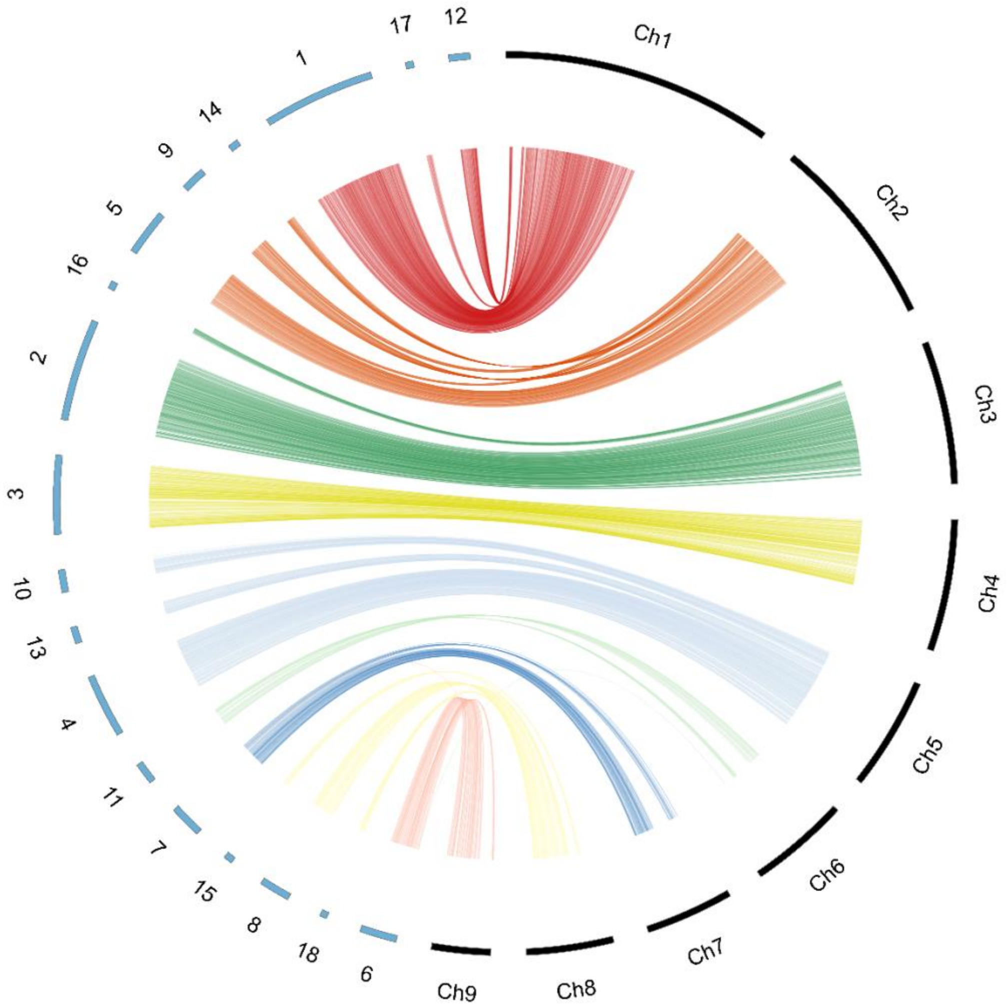

Supplementary Fig. 2. Genome synteny. Synteny of the nine chromosomes (Ch1 to Ch9) of the *Echinococcus granulosus* (genotype G1) genome (*Eg*-G1s) with scaffolds in the previously published genome assembly for *E. granulosus* (genotype G1)[1]. For convenience, unique color was selected for each chromosome to illustrate shared single-copy orthologous genes.

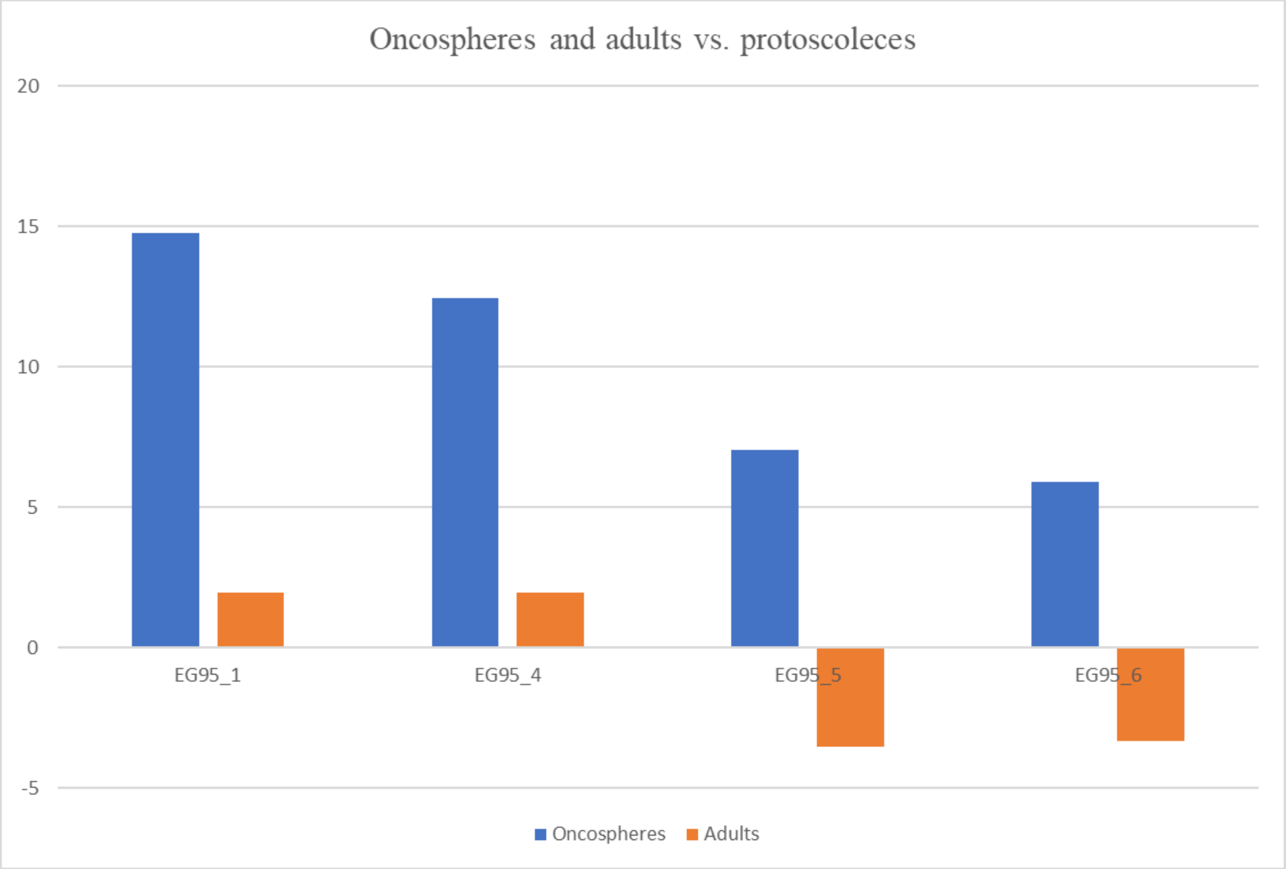

Supplementary Fig. 3. Fold changes (FC; $\log_2$) of EG95 genes among oncosphere, adult and protoscoleces developmental stages. Blue bars represent the FC between the developmental stages of oncosphere and protoscoleces, and orange bar the FC between adult and protoscoleces. The data of FCs are located in Supplementary Table 6.

Supplementary references

1. Tsai, I. J. et al. The genomes of four tapeworm species reveal adaptations to parasitism. *Nature* **496**, 57–63 (2013).
2. Zheng, H. et al. The genome of the hydatid tapeworm *Echinococcus granulosus*. *Nat. Genet.* **45**, 1168–1175 (2013).
