# Supplementary material for: Chromosome-scale Echinococcus granulosus (genotype G1) genome reveals the Eg95 gene family and conservation of the EG95-vaccine molecule
Source: Commun Biol. 2022 Mar 3;5:199. doi: 10.1038/s42003-022-03125-1 (PMC8894454; doi:10.1038/s42003-022-03125-1)
Supplement: Supplementary file 2 — Description of Additional Supplementary Files [file 42003_2022_3125_MOESM2_ESM.pdf]

Description of Additional Supplementary Files

File name: Supplementary Data
Description:

Supplementary Data 1. Protein-coding and non-coding RNA genes in the large repeat regions of the *Echinococcus granulosus* genome (*Eg*-G1s)

Supplementary Data 2. Repeat elements in the *Echinococcus granulosus* genome (*Eg*-G1s)

Supplementary Data 3. Custom repeat families in the *Echinococcus granulosus* genome (*Eg*-G1s)

Supplementary Data 4. Annotation of genes predicted in the *Echinococcus granulosus* genome (*Eg*-G1s)

Supplementary Data 5. Predicted non-coding RNA genes in the *Echinococcus granulosus* genome (*Eg*-G1s)

Supplementary Data 6. Genes predicted for *Echinococcus granulosus* (*Eg*-G1s) which do not have homologues in previous *E. granulosus* genomes (references 14 and 16)

Supplementary Data 7. Genes that were not included in the published set for the *Echinococcus granulosus* genome (*Eg*-G1s)

Supplementary Data 8. Synteny of *Echinococcus granulosus* (*Eg*-G1s) with other cestodes for which high quality genomes are available

Supplementary Data 9. Enriched KEGG pathways in the clusters of correlating genes in *Echinococcus granulosus* (*Eg*-G1s)

Supplementary Data 10. Enriched KEGG BRITE terms in the clusters correlating genes in *Echinococcus granulosus* (*Eg*-G1s)

Supplementary Data 11. Information on the 47 *Echinococcus granulosus* (*Eg*-G1s) samples used in this study and respective short-read sequence data mapped to the four *Eg*95 genes (*Eg*95-1, *Eg*95-4, *Eg*95-5 and *Eg*95-6)

Supplementary Data 12. Variation recorded following the mapping of genomic sequence read data to the four *Eg*95 genes (*Eg*95-1, *Eg*95-4, *Eg*95-5 and *Eg*95-6)

Supplementary Data 13. Average base-contribution of the non-reference base at all variable sites recorded for the four *Eg*95 genes (*Eg*95-1, *Eg*95-4, *Eg*95-5 and *Eg*95-6)

Supplementary Data 14. Positioning of contigs in chromosomes and scaffolds of *Echinococcus granulosus* (*Eg*-G1s)
