# Supplementary material for: Chromosome-scale Echinococcus granulosus (genotype G1) genome reveals the Eg95 gene family and conservation of the EG95-vaccine molecule
Source: Commun Biol. 2022 Mar 3;5:199. doi: 10.1038/s42003-022-03125-1 (PMC8894454; doi:10.1038/s42003-022-03125-1)
Supplement: Supplementary file 4 — Reporting Summary [file 42003_2022_3125_MOESM4_ESM.pdf]

nature portfolio

Corresponding author(s): Prof. Robin Gasser

Last updated by author(s): Nov 25, 2021

Reporting Summary

Nature Portfolio wishes to improve the reproducibility of the work that we publish. This form provides structure for consistency and transparency in reporting. For further information on Nature Portfolio policies, see our Editorial Policies and the Editorial Policy Checklist.

Statistics

For all statistical analyses, confirm that the following items are present in the figure legend, table legend, main text, or Methods section.

n/a	Confirmed	
☐	☒	The exact sample size (*n*) for each experimental group/condition, given as a discrete number and unit of measurement
☒	☐	A statement on whether measurements were taken from distinct samples or whether the same sample was measured repeatedly
☒	☐	The statistical test(s) used AND whether they are one- or two-sided<br>*Only common tests should be described solely by name; describe more complex techniques in the Methods section.*
☒	☐	A description of all covariates tested
☒	☐	A description of any assumptions or corrections, such as tests of normality and adjustment for multiple comparisons
☒	☐	A full description of the statistical parameters including central tendency (e.g. means) or other basic estimates (e.g. regression coefficient) AND variation (e.g. standard deviation) or associated estimates of uncertainty (e.g. confidence intervals)
☒	☐	For null hypothesis testing, the test statistic (e.g. *F*, *t*, *r*) with confidence intervals, effect sizes, degrees of freedom and *P* value noted<br>*Give P values as exact values whenever suitable.*
☒	☐	For Bayesian analysis, information on the choice of priors and Markov chain Monte Carlo settings
☒	☐	For hierarchical and complex designs, identification of the appropriate level for tests and full reporting of outcomes
☒	☐	Estimates of effect sizes (e.g. Cohen's *d*, Pearson's *r*), indicating how they were calculated

Our web collection on statistics for biologists contains articles on many of the points above.

Software and code

Policy information about availability of computer code

Data collection	No software was used.
Data analysis	Described in the text of the manuscript. In summary, following software were used: Trimmomatic v0.36 to filter DNA and RNA-seq reads for quality, Hisat2 v2.1.0 to map reads, Trinity v2.8.4 to assemble RNA-seq data to transcripts, CD-HIT-EST v4.81 to reduce redundancy in assembled RNA-seq transcripts, EdgeR v3.32 to estimatate log2-fold change, RSEM to calculate expected read counts, Assemblosis v0.1.3-beta to create an initial assembly, Purge Haplotigs v1.1.1 to remove redundant sequences, https://gitlab.unimelb.edu.au/vetscience/gapmaster v0.0.1-publication to improve and scaffold contigs in the initial assembly, https://gitlab.unimelb.edu.au/bioscience/annotosis v0.0.1-publication to predict and validate gene models, Infernal v1.1.4 to predict non-coding RNA genes, RepeatModeler v1.0.11 to predict custom repeats, RepeatMasker v4.0.9 to mask the Eg-G1s assembly, OrthoMCL v2.0.4 to predict single copy orthologs (SCOs) for synteny, OrthoFinder v2.5.4 to predict orthologs for Venn diagrams. MrBayes v.3.2.2 and RAxML v.8.0.24 to phylogenetic analysis, DendroPy v.3.12.0 to produce a consensus tree, WGCNA v1.69 to weighted correlation network analysis. SOAPnuke v1.5.6 to filter population genomics DNA read data for quality and verified using FastQC v0.11.8 and MultiQC v1.7, Burrows-Wheeler Aligner (BWA) v.0.7.8 to map read data, mosdepth v.0.3.1 to check read coverage and mapping depth. Genome Analysis Toolkit (GATK) v4.1.3.0 to predict variable sites, Geneious to verify open reading frames (ORF), RoseTTAFold software to predict protein structure.

For manuscripts utilizing custom algorithms or software that are central to the research but not yet described in published literature, software must be made available to editors and reviewers. We strongly encourage code deposition in a community repository (e.g. GitHub). See the Nature Portfolio guidelines for submitting code & software for further information.

Data

Policy information about availability of data

All manuscripts must include a data availability statement. This statement should provide the following information, where applicable:

- Accession codes, unique identifiers, or web links for publicly available datasets
- A description of any restrictions on data availability
- For clinical datasets or third party data, please ensure that the statement adheres to our policy

The nucleotide sequence data from this study are publicly available via the NCBI database: BioProject PRJNA754835 (all genomic and transcriptomic data sets relating to genome Eg-G1s); GenBank accession no. JAIKUZ000000000 (Eg-G1s genome sequence); Sequence Read Archive (SRA) accession nos. SRR15522570, SRR15522571 and SRR15522580 (PacBio long read DNA data for the protoscolex stage of E. granulosus genotype G1); SRR15522572 to SRR15522577, SRR15522581 and SRR15522582 (short-read DNA data for the protoscolex stage of E. granulosus genotype G1); SRR15522578 (RNA-seq data for the oncosphere stage of E. granulosus genotype G1); SRR15522579 (RNA-seq data for the adult stage of E. granulosus genotype G1). GenBank accession nos. MZ889937 to MZ890124 (DNA sequences of each of the four Eg95 genes of each of 47 E. granulosus samples (genotype G1 or G3; derived from short read data).

Field-specific reporting

Please select the one below that is the best fit for your research. If you are not sure, read the appropriate sections before making your selection.

☒ Life sciences ☐ Behavioural & social sciences ☐ Ecological, evolutionary & environmental sciences

For a reference copy of the document with all sections, see nature.com/documents/nr-reporting-summary-flat.pdf

Life sciences study design

All studies must disclose on these points even when the disclosure is negative.

Sample size	Sample sizes are appropriate for the study conducted.
Data exclusions	N/A for this study; no exclusions required.
Replication	N/A for this study; no replication required.
Randomization	N/A for this study; no randomization required.
Blinding	N/A for this study; no blinding required.

Reporting for specific materials, systems and methods

We require information from authors about some types of materials, experimental systems and methods used in many studies. Here, indicate whether each material, system or method listed is relevant to your study. If you are not sure if a list item applies to your research, read the appropriate section before selecting a response.

Materials & experimental systems

n/a	Involved in the study	
☒	☐	Antibodies
☒	☐	Eukaryotic cell lines
☒	☐	Palaeontology and archaeology
☐	☒	Animals and other organisms
☒	☐	Human research participants
☒	☐	Clinical data
☒	☐	Dual use research of concern

Methods

n/a	Involved in the study	
☒	☐	ChIP-seq
☒	☐	Flow cytometry
☒	☐	MRI-based neuroimaging

Animals and other organisms

Policy information about studies involving animals; ARRIVE guidelines recommended for reporting animal research

Laboratory animals	N/A for this study. Not required.
Wild animals	Study did not require or use of wild animals.
Field-collected samples	N/A for this study. Not required.

Ethics oversight	Not required. Described in the manuscript.

Note that full information on the approval of the study protocol must also be provided in the manuscript.
