# Supplementary material for: Chromosome-scale Echinococcus granulosus (genotype G1) genome reveals the Eg95 gene family and conservation of the EG95-vaccine molecule
Source: Commun Biol. 2022 Mar 3;5:199. doi: 10.1038/s42003-022-03125-1 (PMC8894454; doi:10.1038/s42003-022-03125-1)
Supplement: Supplementary file 5 — Supplementary Information [file 42003_2022_3125_MOESM5_ESM.docx]

**Supplementary Table 1 | Sequence read data.** Statistics for the *Echinococcus granulosus* genome (*Eg*-G1s) read data

| Read library | Nucleotide count (bp) | Genome coverage (times) | Number of reads |
| --- | --- | --- | --- |
| PacBio reads | 31,741,108,230 | 212 | 5,247,273 |
| Illumina 250 bp insert paired end reads | 25,128,153,000 | 167 | 251,281,530 |
| RNA-seq reads for Oncospheres | 14,351,109,600 | NA | 143,511,096 |
| RNA-seq reads for Adults | 20,807,757,900 | NA | 208,077,579 |

**Supplementary Table 2 | Repeat content.** Repeat content of the *Echinococcus granulosus* genome (*Eg*-G1s)

| Element | Count | Length occupied (bp) | Percentage of genomic sequence (%) |
| --- | --- | --- | --- |
| Total interspersed repeats | - | 61,416,555 | 35.50 |
| SINEs | 341 | 36,959 | 0.02 |
| LINEs | 2,920 | 169,593 | 0.10 |
| LINE1 | 848 | 52,499 | 0.03 |
| LINE2 | 454 | 24,600 | 0.01 |
| L3/CR1 | 227 | 13,050 | 0.01 |
| LTR elements | 8,247 | 738,603 | 0.43 |
| ERLV | 31 | 1,597 | 0.00 |
| ERLV-MaLRs | 5 | 196 | 0.00 |
| ERV class I | 479 | 24,997 | 0.01 |
| ERV class II | 265 | 13,293 | 0.01 |
| DNA elements | 5,550 | 346,524 | 0.20 |
| hAT-Charlie | 173 | 10,441 | 0.01 |
| TcMar-Tigger | 35 | 2,224 | 0.00 |
| Unclassified | 94,877 | 60,124,876 | 34.76 |
| Small RNA | 2,133 | 303,239 | 0.18 |
| Satellites | 709 | 56,279 | 0.03 |
| Simple repeats | 27,518 | 1,271,317 | 0.73 |
| Low complexity | 3,139 | 147,206 | 0.09 |
|  |  |  |  |
| Total bases masked |  | 62,662,790 | 36.22 |

**Supplementary Table 3 | Frequent repeat elements.** Occurrences of the 20 most frequent repeat elements in the large repeat regions of the *Echinococcus granulosus* genome (*Eg*-G1s)

| Repeat element | Chromosome 1  > 24,500,000 bp | Chromosome 2  > 15,900,000 bp | Chromosome 4  > 12,500,000 bp | Chromosome 7  > 9,400,000 bp | Chromosome 8  > 8,400,000 bp |
| --- | --- | --- | --- | --- | --- |
| DF0000652 | 0 | 0 | 0 | 0 | 62 |
| GA-rich | 0 | 168 | 0 | 52 | 95 |
| rnd-3_family-103 | 432 | 267 | 0 | 0 | 0 |
| rnd-3_family-11 | 0 | 0 | 0 | 0 | 1335 |
| rnd-3_family-165 | 0 | 0 | 389 | 0 | 477 |
| rnd-3_family-167 | 1087 | 847 | 0 | 0 | 0 |
| rnd-3_family-192 | 705 | 400 | 0 | 0 | 0 |
| rnd-3_family-193 | 457 | 301 | 0 | 0 | 0 |
| rnd-3_family-202 | 1004 | 645 | 114 | 0 | 0 |
| rnd-3_family-34 | 0 | 0 | 0 | 162 | 0 |
| rnd-3_family-35 | 0 | 0 | 0 | 171 | 0 |
| rnd-3_family-53 | 0 | 0 | 0 | 211 | 0 |
| rnd-3_family-762 | 0 | 0 | 93 | 0 | 0 |
| rnd-3_family-83 | 0 | 0 | 0 | 0 | 900 |
| rnd-3_family-88 | 0 | 0 | 102 | 0 | 0 |
| rnd-4_family-1125 | 0 | 0 | 83 | 0 | 0 |
| rnd-4_family-116 | 0 | 0 | 155 | 0 | 0 |
| rnd-4_family-1253 | 0 | 0 | 0 | 0 | 63 |
| rnd-4_family-131 | 0 | 0 | 0 | 89 | 0 |
| rnd-4_family-133 | 0 | 0 | 128 | 52 | 0 |
| rnd-4_family-1591 | 0 | 0 | 0 | 0 | 53 |
| rnd-4_family-17 | 556 | 264 | 0 | 0 | 0 |
| rnd-4_family-18 | 390 | 231 | 0 | 0 | 0 |
| rnd-4_family-239 | 0 | 0 | 0 | 0 | 131 |
| rnd-4_family-242 | 0 | 0 | 0 | 0 | 86 |
| rnd-4_family-24 | 340 | 198 | 0 | 0 | 0 |
| rnd-4_family-266 | 0 | 0 | 108 | 0 | 77 |
| rnd-4_family-267 | 0 | 0 | 158 | 0 | 0 |
| rnd-4_family-349 | 200 | 145 | 0 | 0 | 0 |
| rnd-4_family-379 | 0 | 261 | 499 | 0 | 0 |
| rnd-4_family-383 | 0 | 0 | 210 | 0 | 183 |
| rnd-4_family-384 | 0 | 0 | 135 | 0 | 131 |
| rnd-4_family-421 | 0 | 0 | 0 | 0 | 119 |
| rnd-4_family-43 | 0 | 0 | 95 | 0 | 0 |
| rnd-4_family-469 | 0 | 0 | 85 | 0 | 0 |
| rnd-4_family-470 | 0 | 0 | 162 | 0 | 0 |
| rnd-4_family-49 | 0 | 0 | 0 | 189 | 0 |
| rnd-4_family-616 | 0 | 187 | 0 | 0 | 54 |
| rnd-4_family-723 | 369 | 201 | 0 | 0 | 0 |
| rnd-4_family-765 | 0 | 0 | 0 | 127 | 0 |
| rnd-4_family-770 | 855 | 638 | 0 | 0 | 0 |
| rnd-4_family-858 | 411 | 262 | 0 | 0 | 0 |
| rnd-4_family-971 | 0 | 0 | 150 | 0 | 0 |
| rnd-4_family-97 | 383 | 223 | 0 | 0 | 0 |
| rnd-5_family-12 | 249 | 197 | 0 | 0 | 90 |
| rnd-5_family-1239 | 326 | 221 | 0 | 0 | 0 |
| rnd-5_family-13 | 444 | 339 | 0 | 0 | 0 |
| rnd-5_family-1394 | 0 | 0 | 0 | 0 | 71 |
| rnd-5_family-1395 | 0 | 0 | 0 | 0 | 119 |
| rnd-5_family-1521 | 0 | 0 | 0 | 0 | 91 |
| rnd-5_family-2109 | 201 | 0 | 0 | 0 | 0 |
| rnd-5_family-2153 | 0 | 0 | 0 | 99 | 0 |
| rnd-5_family-217 | 0 | 0 | 100 | 0 | 0 |
| rnd-5_family-221 | 0 | 0 | 0 | 81 | 0 |
| rnd-5_family-223 | 0 | 0 | 0 | 81 | 0 |
| rnd-5_family-2438 | 0 | 0 | 0 | 57 | 0 |
| rnd-5_family-245 | 0 | 0 | 0 | 0 | 95 |
| rnd-5_family-2676 | 0 | 0 | 0 | 0 | 53 |
| rnd-5_family-389 | 0 | 0 | 0 | 51 | 0 |
| rnd-5_family-4054 | 0 | 0 | 0 | 53 | 0 |
| rnd-5_family-593 | 294 | 271 | 239 | 87 | 0 |
| rnd-5_family-633 | 0 | 0 | 110 | 0 | 0 |
| rnd-5_family-650 | 0 | 0 | 0 | 54 | 0 |
| rnd-5_family-716 | 0 | 0 | 0 | 100 | 0 |
| rnd-5_family-718 | 0 | 0 | 0 | 75 | 0 |
| rnd-5_family-815 | 253 | 0 | 0 | 0 | 0 |
| rnd-5_family-853 | 0 | 0 | 0 | 54 | 0 |
| rnd-5_family-962 | 0 | 0 | 0 | 116 | 0 |

**Supplementary Table 4 | Gene copy numbers.** Differences in the copy number (n ≥ 5) of the predicted genes among three *Echinococcus granulosus* genomes (i.e. *Eg*-G1s reference and two draft genomes published previously^1,2^

| Orthogroup ID | Gene count in *Eg*-G1 | Gene count in *Eg*^a^ | Gene count in *Eg*^b^ | Total gene count in orthogroup | Description |
| --- | --- | --- | --- | --- | --- |
| OG0000000 | 69 | 2 | 3 | 74 | Histone H2A^c^ |
| OG0000001 | 68 | 2 | 2 | 72 | Histone H2B^c^ |
| OG0000002 | 59 | 0 | 0 | 59 | Variant Surface Glycoprotein^c^ |
| OG0000003 | 33 | 0 | 2 | 35 | Histone H4^c^ |
| OG0000004 | 19 | 1 | 1 | 21 | Histone H2A^c^ |
| OG0000005 | 16 | 1 | 0 | 17 | Hypothetical protein^d^ |
| OG0000006 | less | 1 | 0 | 17 | Inosine triphosphate pyrophosphatase^c^ |
| OG0000007 | 0 | 5 | 11 | 16 | Retrovirus-related Pol polyprotein^e^ |
| OG0000008 | 10 | 3 | 2 | 15 | RNA helicase |
| OG0000009 | 13 | 0 | 0 | 13 | Origin recognition complex subunit^c^ |
| OG0000010 | 11 | 0 | 1 | 12 | Mucin-22 like protein^c^ |
| OG0000011 | 9 | 1 | 1 | 11 | Hypothetical protein^c^ |
| OG0000012 | 6 | 3 | 1 | 10 | Multidrug andc toxin extrusion protein^c^ |
| OG0000013 | 8 | 1 | 1 | 10 | Inosine triphosphate pyrophosphatase^c^ |
| OG0000014 | 6 | 1 | 3 | 10 | Hypothetical protein^f^ |
| OG0000016 | 7 | 1 | 1 | 9 | Ureohydrolase |
| OG0000019 | 9 | 0 | 0 | 9 | Origin recognition complex subunit^f^ |
| OG0000020 | 8 | 1 | 0 | 9 | Hypothetical protein^f^ |
| OG0000022 | 0 | 2 | 6 | 8 | Hypothetical protein |
| OG0000023 | 0 | 3 | 5 | 8 | Retrotransposon-derived protein^e^ |
| OG0000026 | 6 | 1 | 1 | 8 | Hypothetical protein^c^ |
| OG0000027 | 6 | 1 | 1 | 8 | Hypothetical protein |
| OG0000029 | 5 | 3 | 0 | 8 | Cysteine protease |
| OG0000030 | 0 | 0 | 7 | 7 | Hypothetical protein |
| OG0000037 | 0 | 5 | 2 | 7 | Heat shock protein |
| OG0000047 | 7 | 0 | 0 | 7 | Rho GTPase activation protein |
| OG0000049 | 0 | 1 | 5 | 6 | Retrovirus-related Pol polyprotein^e^ |
| OG0000051 | 0 | 1 | 5 | 6 | Hypothetical protein |
| OG0000058 | 0 | 0 | 6 | 6 | Hypothetical protein |
| OG0000061 | 0 | 5 | 1 | 6 | Hypothetical protein |
| OG0000066 | 5 | 0 | 1 | 6 | DNA replication licensing factor |
| OG0000080 | 6 | 0 | 0 | 6 | Neurofilament heavy polypeptide |
| OG0000081 | 6 | 0 | 0 | 6 | Phosphotidylinositol phosphatase |
| OG0000082 | 5 | 1 | 0 | 6 | Hypothetical protein |
| OG0000089 | 0 | 0 | 5 | 5 | Hypothetical protein |
| OG0000237 | 5 | 0 | 0 | 5 | Hypothetical protein^f^ |

^a^Short read *Echinococcus granulosus* assembly^1^

^b^Short read *Echinococcus granulosus* assembly^2^

^c^Located mainly in repeat regions of *Eg*-G1s

^d^Homologous to EmuJ_000357400 in *Echinococcus multilocularis*

^e^Putative transposons

^f^Located partially in repeat regions of *Eg*-G1s

**Supplementary Table 5 | *Eg*-95 transcription.** Differential transcription of Eg95 genes in *Echinococcus granulosus* genome (*Eg*-G1s)

| Gene annotation | Gene ID | log_2_ FC |
| --- | --- | --- |
| Oncospheres vs. protoscoleces |  |  |
| EG95-1 | ECG_03412 | 15.55 |
| EG95-4 | ECG_06032 | 12.2756 |
| EG95-5 | ECG_02114 | 6.58807 |
| EG95-6 | ECG_02112 | 5.48484 |
| Adults vs. protoscoleces |  |  |
| EG95-1 | ECG_03412 | 15.55 |
| EG95-4 | ECG_06032 | 12.2756 |
| EG95-5 | ECG_02114 | 6.58807 |
| EG95-6 | ECG_02112 | 5.48484 |


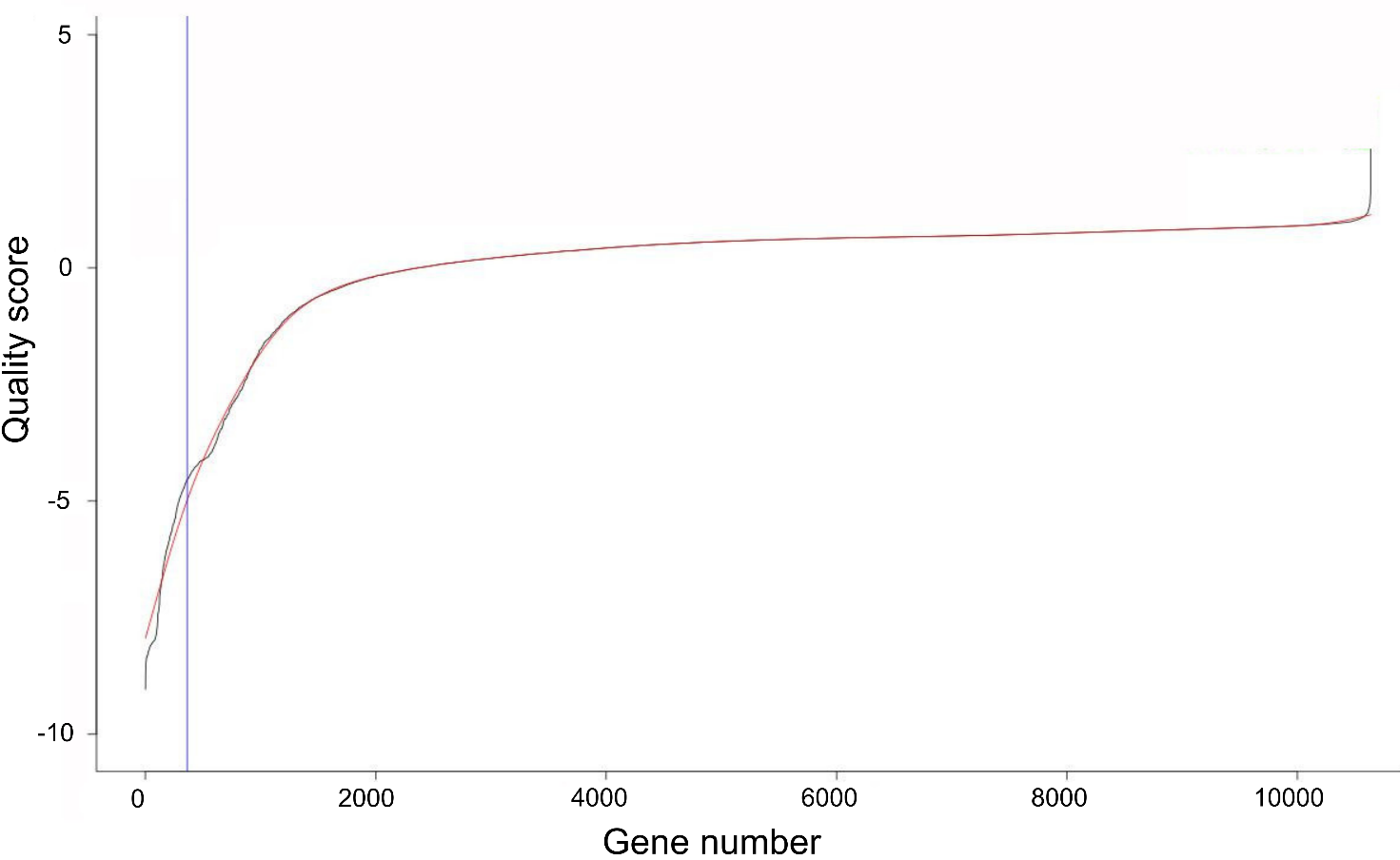


**Supplementary Fig. 1.** **Scoring the quality of predicted genes.** The graph displays the quality scores for 10,638 genes, in which a higher quality score reflects a more confident gene prediction. A black curve displays the calculated quality scores for individual genes. The smoothened red curve was used to estimate the cut-off (blue vertical line) for a low quality gene prediction (left of the line). The cut-off was set at the steepest curvature value of the red curve before the shoulder point.

**
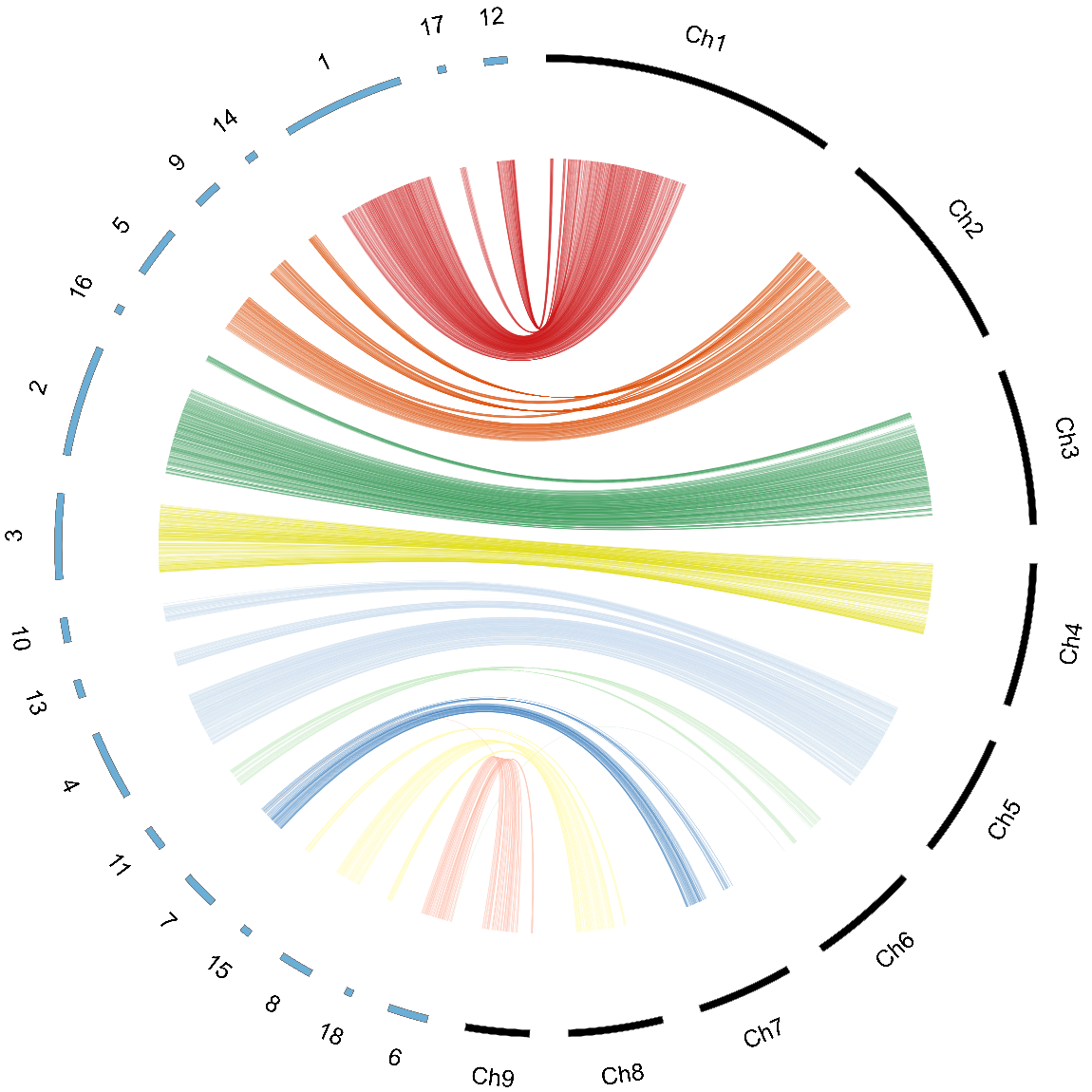
**

**Supplementary Fig. 2.** **Genome synteny.** Synteny of the nine chromosomes (Ch1 to Ch9) of the *Echinococcus granulosus* (genotype G1) genome (*Eg-*G1s) with scaffolds in the previously published genome assembly for *E. granulosus* (genotype G1)^1^. For convenience, unique color was selected for each chromosome to illustrate shared single-copy orthologous genes.


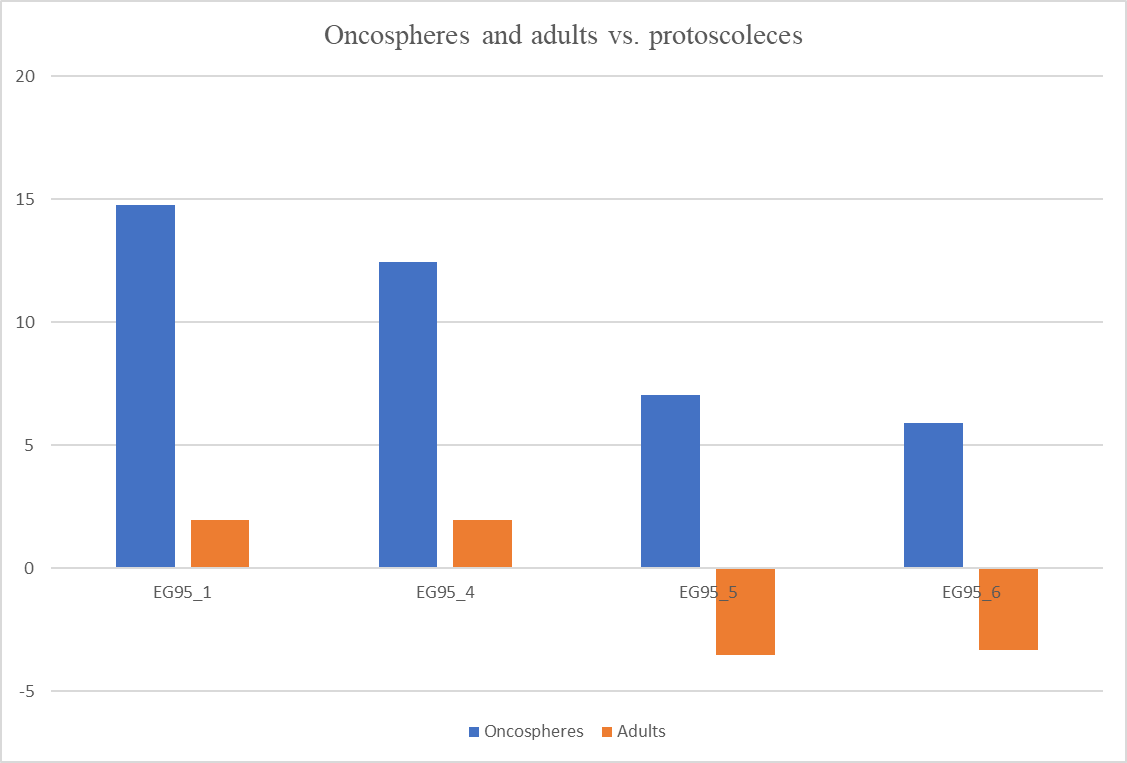


**Supplementary Fig. 3.** **Fold changes (FC; log_2_) of EG95 genes among oncosphere, adult and protoscoleces developmental stages.** Blue bars represent the FC between the developmental stages of oncosphere and protoscoleces, and orange bar the FC between adult and protoscoleces. The data of FCs are located in Supplementary Table 6.

**Supplementary references**

1. Tsai, I. J. et al. The genomes of four tapeworm species reveal adaptations to parasitism. *Nature* **496**, 57–63 (2013).
2. Zheng, H. et al. The genome of the hydatid tapeworm *Echinococcus granulosus*. *Nat. Genet.* **45**, 1168–1175 (2013).
